# Inhibition of inflammatory CCR2 signaling promotes aged muscle regeneration and strength recovery after injury

**DOI:** 10.1038/s41467-020-17620-8

**Published:** 2020-08-20

**Authors:** Roméo S. Blanc, Jacob G. Kallenbach, John F. Bachman, Amanda Mitchell, Nicole D. Paris, Joe V. Chakkalakal

**Affiliations:** 1 Department of Pharmacology and Physiology, University of Rochester Medical Center, Rochester, NY USA; 2 Wilmot Cancer Institute, Stem Cell and Regenerative Medicine Institute, and The Rochester Aging Research Center, University of Rochester Medical Center, Rochester, NY USA; 3 Department of Biomedical Engineering, University of Rochester Medical Center, Rochester, NY USA; 4 Department of Pathology and Laboratory Medicine, Cell Biology of Disease Graduate Program, University of Rochester Medical Center, Rochester, NY USA

**Keywords:** Ageing, Chemokines, Muscle stem cells, Regeneration

## Abstract

Muscle regeneration depends on a robust albeit transient inflammatory response. Persistent inflammation is a feature of age-related regenerative deficits, yet the underlying mechanisms are poorly understood. Here, we find inflammatory-related CC-chemokine-receptor 2 (Ccr2) expression in non-hematopoietic myogenic progenitors (MPs) during regeneration. After injury, the expression of Ccr2 in MPs corresponds to the levels of its ligands, the chemokines Ccl2, 7, and 8. We find stimulation of Ccr2-activity inhibits MP fusion and contribution to myofibers. This occurs in association with increases in MAPKp38δ/γ signaling, MyoD phosphorylation, and repression of the terminal myogenic commitment factor Myogenin. High levels of Ccr2-chemokines are a feature of regenerating aged muscle. Correspondingly, deletion of *Ccr2* in MPs is necessary for proper fusion into regenerating aged muscle. Finally, opportune Ccr2 inhibition after injury enhances aged regeneration and functional recovery. These results demonstrate that inflammatory-induced activation of Ccr2 signaling in myogenic cells contributes to aged muscle regenerative decline.

## Abstract (web summary)

Chronic inflammation is a feature of age-related regenerative decline in skeletal muscles, but how it directly affects resident muscle stem cell fate and function is unclear. Here, the authors show that Ccr2 signaling in muscle stem cell derived progenitors represses terminal myogenic differentiation, and that targeting Ccr2 on aged myogenic progenitors rejuvenates aged skeletal muscle healing and function.

## Introduction

During tissue regeneration, the recruitment of inflammatory cells is a critical early response to injury. This recruitment aids in the establishment of a favorable environment for progenitor function and tissue regeneration^1,2^. Chemokines play an important role in the recruitment of inflammatory cells to sites of injury; however, persistently elevated signaling contributes to chronic inflammation associated with impaired regeneration^2–7^. Among the large chemokine superfamily members, Ccl2, 7, and 8 bind a shared receptor, Ccr2, and have key roles in the deleterious consequences of chronic chemokine activity^4,6–15^. As such, inhibition of Ccr2 is being pursued as a clinical therapy in disease contexts^16–19^. As a G-protein coupled transmembrane receptor, ligand-mediated activation of Ccr2 mobilizes intracellular G-proteins that help activate several pathways, including Erk and p38Mapk^3,20,21^. Abnormal activity in these intracellular mediators has been implicated in age-related stem and progenitor cell dysfunction^22^. A role for Ccr2 has also been described during skeletal muscle regeneration^4,6,23–26^. Mice germ-line deficient in *Ccr2*—or the gene encoding for its primary cognate ligand *Ccl2*—phenocopy the adverse effects of macrophage and monocyte ablation during the early stages of muscle regeneration^24,27^. However, in the chronic muscle degenerative disorder Duchenne muscular dystrophy, Ccr2 genetic or pharmacological inhibition has transient benefits through modulation of inflammatory cell populations and do not exacerbate pathology^4,6,28^.

The regenerative capacity of skeletal muscle relies on a population of non-hematopoietic Pax7-expressing muscle stem cells called satellite cells (SCs)^29^. In adults, SCs reside in a primarily quiescent state^30–32^. In response to a degenerative insult, SCs activate, proliferate, differentiate, and the derived progenitor cells fuse to form multinucleated muscle fibers (myofibers); thus, fulfilling skeletal muscle regeneration^1,29^. Analogous to other tissues and organs, the regenerative potential of skeletal muscle declines with age^33,34^. Skeletal muscle is a critical effector for movement and a key regulator of whole-body metabolism; hence, delays in skeletal muscle recovery from injury often observed in the elderly severely impacts activities of daily living, quality of life, and risk for falling and fractures. Although features of this decline include loss of SC number and function, a sub-population persists with a regenerative potential that can be stimulated^22,31,32^. Therefore, understanding the processes leading to functional decline within this sub-population of SC and derived progenitor cells is essential to promote aged skeletal muscle regeneration, strength recovery, and thereby healthy aging.

The role of Ccr2 in non-hematopoietic cells is largely understudied, especially in the context of tissue regeneration and aging. Although Ccr2 signaling can influence the fate of immortalized C2C12 myoblasts^25^, whether Ccr2 can directly influence SC and derived progenitor fate and function remains to be elucidated. Here, we demonstrate that *Ccr2* is expressed in active SCs and myogenic progenitors (MPs) derived from regenerating skeletal muscles. At high levels, Ccr2 chemokines stimulate mitogen activated protein kinases p38δ/γ activity, MyoD phosphorylation, and downstream repression of the terminal myogenic commitment factor Myogenin. This was associated with the ability of high Ccr2 chemokine levels either through supplementation or in the context of aging to inhibit MP fusion and multinucleated myofiber formation. Finally, we observe that targeting elevated Ccr2 activity at later stages of recovery promotes aged muscle regeneration and recuperation of strength following an acute muscle injury.

## Results

### Activated SCs and MPs from regenerating muscle express Ccr2

**Fig. 1:**
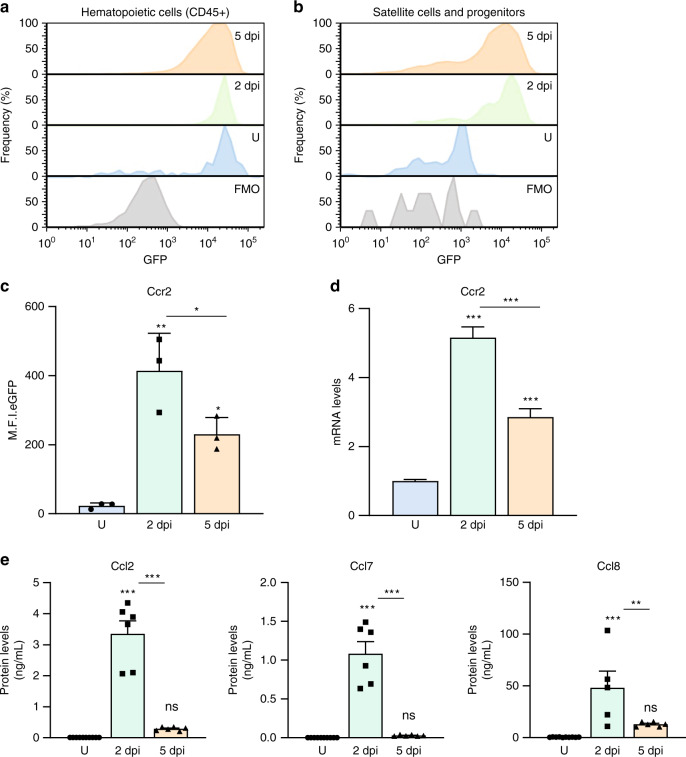
Ccr2 expression in activated SCs and MPs from regenerating muscle. **a**, **b** Representative FACS histograms of Ccr2 reporter fluorescence (eGFP) in **a** hematopoietic cells (CD45+), and **b** SCs and MPs (Lin- β1Int + VCAM+) from adult (2 months old) mouse skeletal muscles uninjured (U) and injured muscles, 2- and 5-days post injury (2dpi and 5dpi) (*n* = 3 mice per condition). **c** Mean fluorescence intensity of eGFP (Ccr2) in SCs and MPs from adult uninjured and injured limb muscles, 2- and 5-days post injury (2dpi and 5dpi) (*n* = 3 mice per condition). **d** Expression of *Ccr2* in SCs and MPs from adult uninjured and injured limb muscles, 2- and 5-days post injury (2dpi and 5dpi) (*n* = 3 mice per condition). **e** Levels of inflammatory Ccr2 chemokine ligands (Ccl2, Ccl7 and Ccl8) from adult uninjured, 2dpi and 5dpi muscles with multiplex Luminex assay (*n* = 6 mice per condition). Flow cytometry data are reported as mean ± s.e.m. from at least 500000 events per acquisition. Cells were gated for single cells and live cells (DAPI) prior to specific cell lineage markers (Supplementary Fig. S1) and GFP gating. mRNA levels are reported as fold-change ± s.d. relative to *Gapdh* and *B2m* and normalized to experimental control. Luminex data are reported as protein concentration from muscle lysates (mean ± s.e.m., ng/mL). Non-significant (n.s.) *P* > 0.05, *P < 0.05, **P < 0.01, ***P < 0.001, non-parametric one-way ANOVA followed by Tukey’s multiple comparisons test. * is relative to uninjured except if otherwise indicated (bar).

To examine *Ccr2* expression during muscle regeneration we used mice heterozygous for an eGFP reporter in the *Ccr2* coding region (Ccr2^GFP/+^)^26,35,36^. Reporter activity was evaluated in hematopoietic cells (CD45+), and satellite cells (SCs) (Lin−, Sca1−, Vcam+, β1Int+) isolated from adult (2-months-old mice) uninjured, 2 days post-degenerative injured (2dpi), and 5dpi Ccr2^GFP/+^ skeletal muscles (Supplementary Fig. 1a)^34^. Consistent with previous reports, we found that CD45 positive (CD45+) cells express high levels of Ccr2 (Fig. 1a)^23,24,37^. Although *Ccr2* reporter expression is negligible in SCs from uninjured muscle, we found relatively high levels in active SCs and MPs from 2dpi, and to a lesser extent, 5dpi muscle (Fig. 1b, c). This pattern of *Ccr2* expression was also observed in active SCs and MPs isolated from regenerating muscle at the mRNA level (Fig. 1d). In addition, we confirmed the presence of Ccr2 receptor on MyoD+ MPs (Supplementary Fig. 1b). Thus, Ccr2 is expressed in activated SCs and MPs from regenerating muscle.

To determine if *Ccr2* expression in active SCs and MPs correspond to the presence of ligands in regenerating muscle, we assessed the levels of Ccr2 cognate ligands Ccl2, Ccl7, and Ccl8 in uninjured, 2dpi and 5dpi skeletal muscles. Both mRNA and protein levels of the Ccr2 chemokines increased at 2dpi and decreased at 5dpi (Fig. 1e and Supplementary Fig. 2a). Therefore, the levels of *Ccr2* in active SCs and MPs correspond to the levels of Ccr2-ligands in the adult regenerative milieu. We next asked whether the decrease in Ccr2-ligands from a peak at 2dpi could account for the reduction of *Ccr2* expression in active SCs and MPs at 5dpi. To address this, we systemically delivered a cocktail of Ccr2-ligands (recombinant Ccl2, Ccl7 and Ccl8; Ccl^2/7/8^) to Ccr2^GFP/+^ mice 12 h prior to flow cytometry analysis of SCs and MPs from 5dpi muscle (Supplementary Fig. 2b, c). We found Ccl^2/7/8^ supplementation was sufficient to increase *Ccr2* expression in active SCs and MPs from 5dpi adult muscles (Supplementary Fig. 2c, d). To investigate whether Ccr2 chemokines can regulate *Ccr2* expression, sorted SCs from wildtype (Ccr2^+/+^) and Ccr2 deficient (Ccr2^−/−^) mice were cultured and derived MPs were treated with different concentrations of Ccl^2/7/8^ (Supplementary Fig. 3). In response to Ccl^2/7/8^ supplementation, both Ccr2 mRNA and protein levels increased in a dose-dependent manner in Ccr2^+/+^ SC cultures, while Ccr2 expression was essentially undetectable in Ccr2^−/−^ SC cultures (Supplementary Fig. 3a, b). Therefore, Ccr2 levels can be enhanced by its own ligands.

### Ccr2 signaling inhibits terminal myogenic differentiation

**Fig. 2:**
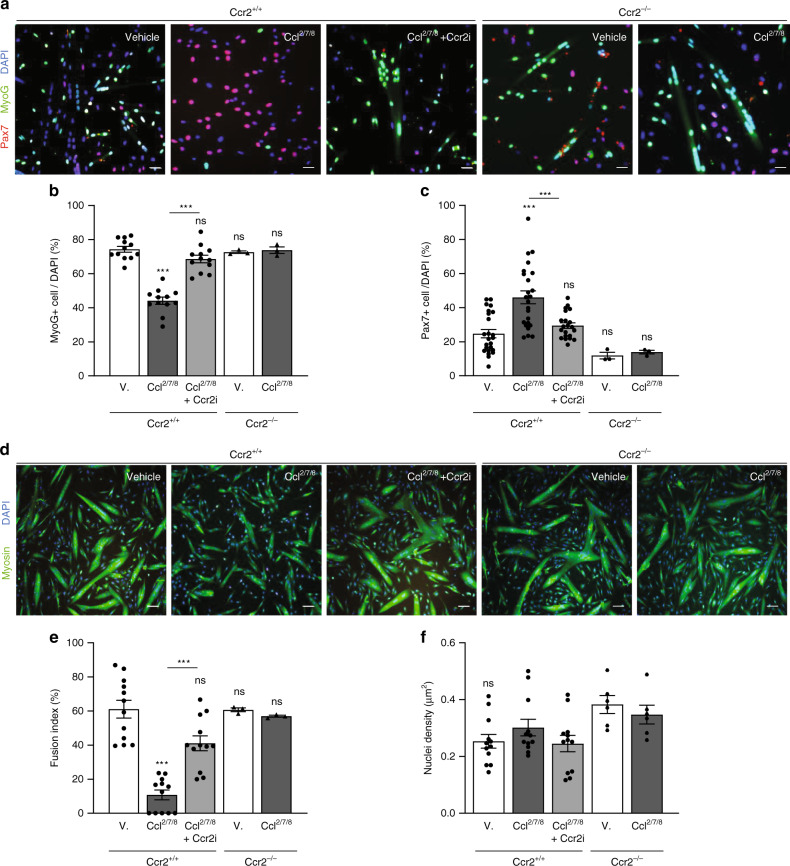
Ccr2 chemokines prevent SC-derived MP terminal commitment and fusion. **a** Representative images from immunofluorescence staining of SC cultures derived from adult (2 months old) wildtype (Ccr2^+/+^) and Ccr2-null mutant (Ccr2^−/−^) mice. Cells were purified by MACS and, 5000 SCs were plated and cultured for 96 h in growing media prior to low serum condition media (differentiation media; DM) supplemented with vehicle (V), Ccr2-ligands (Ccl^2/7/8^) or Ccl^2/7/8^ and Ccr2 small inhibitor (Ccr2i) for 24 hours. Red, Pax7 (SC); Green, MyoG (Differentiating MP); Blue, DAPI (Nuclei). Experiments were performed at least three times. **b**, **c** Percentage of **b** MyoG+ and **c** Pax7+ cells of total cells (DAPI+) per culture of SCs derived from adult Ccr2^+/+^ (*n* = 12) and Ccr2^−/−^ (*n* = 3) mice. Each dot represents the percentage of positive cells averaged from duplicate cultures from one mouse (*n* = 3–12 mice). **d** Representative images from immunofluorescence staining of SC cultures derived from adult wildtype Ccr2^+/+^ and Ccr2^−/−^ mice. Cells were purified by MACS and 10000 cultured for differentiation and myotube formation in DM supplemented with vehicle, Ccl^2/7/8^ or Ccl^2/7/8^ and Ccr2i for 24 hours. Green, Myosin (Myotubes); Blue, DAPI (Nuclei). Experiments were performed at least three times. **e**, **f** MP cultures **e** fusion index and **f** cell density (DAPI + /um^2^). Fusion index was measured by number of fused myonuclei within myotubes relative to total number of cells (DAPI) per culture of SCs derived from adult Ccr2 + /+ (*n* = 12) and Ccr2−/− (*n* = 3) mice. Each dot is the averaged data from duplicate cultures from one mouse. Culture data are percentage mean ± s.e.m. For each experiment, cells were derived from one mouse and cultured into at least two wells per condition and >300 cells were counted. Scale bars, (a) 20 μm, (d) 50 μm. Non-significant (n.s.) *P* > 0.05, *P < 0.05, **P < 0.01, ***P < 0.001, non-parametric two-way ANOVA followed by Tukey’s multiple comparisons test. Stats are relative to Vehicle except if otherwise indicated (bar).

Next, we examined if Ccr2 can function as a regulator of active SC and MP fate. To test this, isolated SCs were cultured in growth media for 48 h, and subsequently supplemented for 24 h with vehicle or Ccl^2/7/8^. Regardless of treatment, all cells were MyoD+ (MP marker); however, Ccl^2/7/8^ treatment decreased the proportion of Myogenin+ (MyoG+, terminal myogenic commitment/fusion marker) cells without affecting cell density (Supplementary Fig. 4a–d). Importantly, we did not observe a significant effect of Ccr2-ligands on proliferation (EdU+ cells; Supplementary Fig. 4e, f). These data suggest Ccr2 regulates terminal myogenic commitment. To assess the effect on fusion, we cultured isolated wildtype and Ccr2^−/−^ SCs at a high density for 96 h in growth media, and subsequently switched to low serum media that promotes fusion for 24 h supplemented with combinations of vehicle, Ccl^2/7/8^, or a Ccr2 small molecule inhibitor BMS CCR2 22 (Ccr2i). Ccr2i is a competitive binding inhibitor with a selective and high affinity for Ccr2’s binding pocket, rendering the receptor inactive albeit stable^38^. Treatment with Ccl^2/7/8^ reduced the proportion of Ccr2^+/+^ cells expressing MyoG (Fig. 2a, b). This was coupled to a higher proportion of Pax7+ (SC renewal marker) cells (Fig. 2a, c). The capacity for Ccl^2/7/8^ to reduce the proportion of MyoG+ in favor of Pax7+ cells was reversed by the addition of Ccr2i and in Ccr2^−/−^ cells (Fig. 2a–c). Next, we quantified the number of nuclei incorporated into skeletal muscle myosin positive myotubes (fusion index). Consistent with the loss of fusion-competent MyoG+ cells (Fig. 2a, b), Ccl^2/7/8^ treatment impaired myotube formation based on reduced fusion index (Fig. 2d, e). Importantly, Ccl^2/7/8^ treatment was unable to impair myotube formation in the presence of Ccr2i, or when Ccr2 was deficient (Fig. 2d, e). Regardless of treatment or genotype, Ccl^2/7/8^ treatment did not affect nuclear density (Fig. 2f). Therefore, Ccr2 is dispensable for aspects of active SC and MP function devoted to cell growth^24,39^. However, in the presence of high ligand levels, Ccr2 can directly inhibit MP fusion capacity and the formation of multinucleated myotubes.

**Fig. 3:**
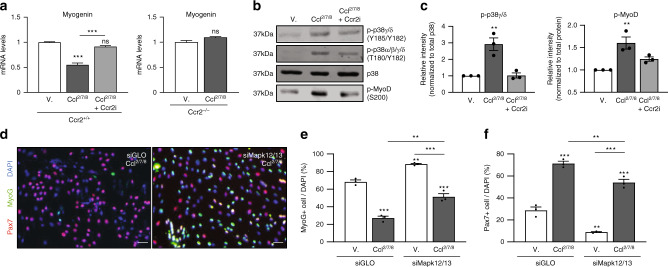
Ccr2 represses *Myogenin* expression through p38δγ signaling. **a** *Myogenin* mRNA expression in SCs derived myotubes from adult (2 months old) wildtype (Ccr2^+/+^) (*n* = 6) and Ccr2-null mutants (Ccr2^−/−^) (*n* = 3) mice treated with vehicle (V), Ccr2-ligands (Ccl^2/7/8^) or Ccl^2/7/8^ and Ccr2 small inhibitor (Ccr2i) for 24 h. **b**, **c** Representative immunoblotting from treated SC derived myotubes (**b**) and **c** quantification of phospho-p38δγ normalized to total p38, and of phospho-MyoD normalized to total protein (*n* = 3 mice). **d** Immunofluorescence staining of adult SC derived cultures treated with siRNAs against *Mapk12/13* or siGLO (control) after 72 h then directed toward differentiation at 96 h in DM supplemented with vehicle or Ccl^2/7/8^ for 24 h. Pax7 (Red); MyoG (Green); DAPI (Blue). Experiments were performed at least three times. **e**, **f** Percentage of **e** MyoG+ MPs and **f** Pax7+ SCs normalized to total number of cells (DAPI+). Each dot represents the percentage of positive cells averaged from duplicate cultures from one mouse (*n* = 3 mice). RTqPCR analysis was performed in triplicate from at least two animal per experiment and condition. mRNA levels are reported as fold-change ± s.d. relative to *Gapdh* and *B2m* and normalized to experimental control. Immunoblots were performed from lysates derived from at least two animal per condition from at least three experiments. Data are mean ± s.e.m. For each experiment we plated cells from one mouse into at least two wells per condition and used at least three mice per group and counted >400 cells. siRNA experiments were pre-validated using two different siRNAs for each gene target and efficient siRNAs were controlled for off-target effects toward other p38MAPK gene members (Supplementary Fig. S5e). Scale bars, **d** 25 μm. n.s. not significant, *P < 0.05, **P < 0.01, ***P < 0.001, non-parametric one-way ANOVA followed by Tukey’s multiple comparisons test. Asterisk is relative to vehicle from sample condition except if otherwise indicated by a line.

To test whether Ccl^2/7/8^-mediated reduction in the proportion of MyoG+ cells in differentiating conditions involves transcriptional repression, we examined *Myogenin* expression. Indeed, treatment of differentiating SC cultures with Ccl^2/7/8^ resulted in reduced *Myogenin* expression (Fig. 3a). The Ccl^2/7/8^-mediated repression of *Myogenin* expression was prevented with Ccr2i treatment or Ccr2 deficiency (Ccr2^−/−^) (Fig. 3a). Given that Ccr2 activity inhibits *Myogenin* expression, we investigated downstream signals potentially mediating this effect^40^. Individual chemokines induced rapid phosphorylation of the critical intracellular signaling mediators Erk and p38MAPK, which are known regulators of the myogenic differentiation program (Supplementary Fig. 5a, b)^31,41–44^. Among these, we found Ccl^2/7/8^ treatment induced prolonged and enhanced activation of p38MAPK signaling (Supplementary Fig. 5b). The p38MAPK family is composed of four distinct isoforms encoded by four genes often associated by pair due to their similarity and overlapping function across various tissues^45–49^. In this regard, previous reports show that p38α/β promote, while p38δ/γ inhibit terminal myogenic commitment^42–44^. Inhibition of myogenic differentiation by activated p38δ/γ involves phosphorylation of MyoD on serine 200 and repression of *Myogenin* expression^44^. Consistent with the ability to repress *Myogenin* expression, Ccl^2/7/8^ increased phosphorylated levels of p38δ/γ and MyoD on serine 200 in a Ccr2-dependent manner (Fig. 3b, c). Furthermore, both Ccl^2/7/8^-mediated repression of *Myogenin* expression and p38δ/γ activation were dosage dependent (Supplementary Fig. 5c, d). Subsequently, we sought to determine if p38δ/γ siRNAs (*siMapk12*/*13*) could circumvent Ccr2-mediated loss of MyoG+ cells (Supplementary Fig. 5e). Consistent with p38δ/γ genes knockdown as a means to promote terminal myogenic commitment, treatment of cultured SCs with *siMapk12/13* increased and decreased the proportion of MyoG+ and Pax7+ cells, respectively (Fig. 3d–f). In response to Ccl^2/7/8^ supplementation, *siMapk12/13* treatment attenuates the loss of MyoG+ cells (Fig. 3d–f). Thus, p38δ/γ signaling contributes to Ccr2-mediated inhibition of MP progression toward fusion competency necessary for myofiber formation.

**Fig. 4:**
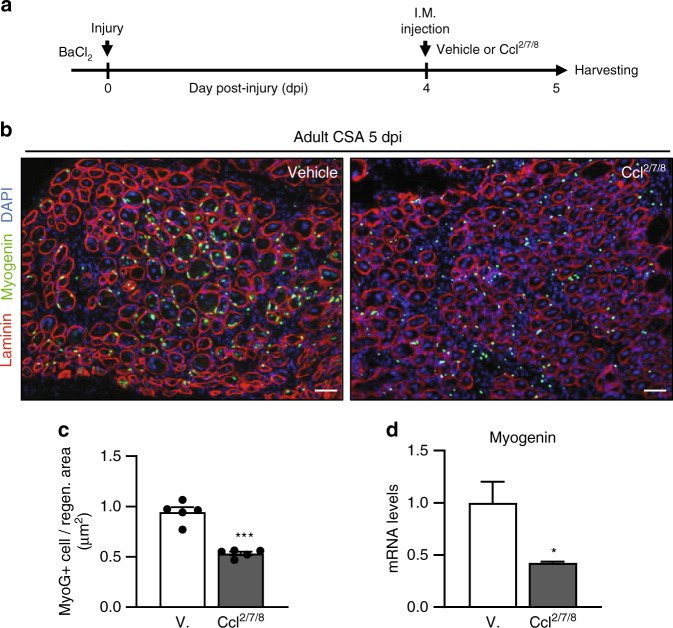
Prolonged Ccr2 signaling during adult muscle regeneration negatively affects myogenesis. **a** Strategy to extend Ccr2-ligands (Ccl^2/7/8^) presence in regenerating muscles. Adult (2 months old) mice were injured with intramuscular (IM) injection of barium chloride, followed by IM injection of recombinant chemokines or saline (vehicle) and harvested for analysis at 5dpi. **b** Immunofluorescence staining of the injured muscles from adult mice treated with vehicle (*n* = 5) or Ccl^2/7/8^ (*n* = 5). Laminin (red); MyoG (green); DAPI (blue). **c** Percentage of MyoG+ MPs per area of regeneration (regen. area; μm^2^) from adult injured muscles at 5dpi following treatment (*n* = 5 mice per condition). **d** v*Myogenin* transcript levels in freshly isolated SCs and MPs from adult injured muscles (5dpi) following chemokines treatment (*n* = 3 mice per condition). Data are reported as mean ±  s.e.m. Scale bars, 100 μm. *P < 0.05, **P < 0.01, ***P < 0.001, non-parametric one-way ANOVA followed by Tukey’s multiple comparisons test.

Five days after degenerative injury to adult muscle, we found Ccr2 chemokines expression had returned to near uninjured baseline levels (Fig. 1e, and Supplementary Fig. 2). At this time, adult regenerating muscle contains a high number of fusion-competent MyoG+ progenitors (Fig. 4b, green). Since Ccr2 chemokines can inhibit *Myogenin* expression, we tested if Ccr2 chemokines treatment to regenerating adult muscle can lead to a reduction in MyoG+ progenitors. To this end, we locally delivered vehicle or Ccl^2/7/8^ at 4dpi and subsequently examined muscles at 5dpi for MyoG+ progenitor number (Fig. 4a, b). We found adult regenerating muscle exposed to Ccr2-ligands had reduced MyoG+ cells compared to vehicle (Fig. 4b, c). Next, we sought to determine if the loss of MyoG+ cells reflects in part reduced *Myogenin* expression in active SCs and MPs. Examination of SCs and progenitors derived from 5dpi muscles revealed that Ccl^2/7/8^ treatment in vivo reduced *Myogenin* expression by over 50% (Fig. 4d).

### Deletion of Ccr2 promotes adult MP fusion in aged muscle

**Fig. 5:**
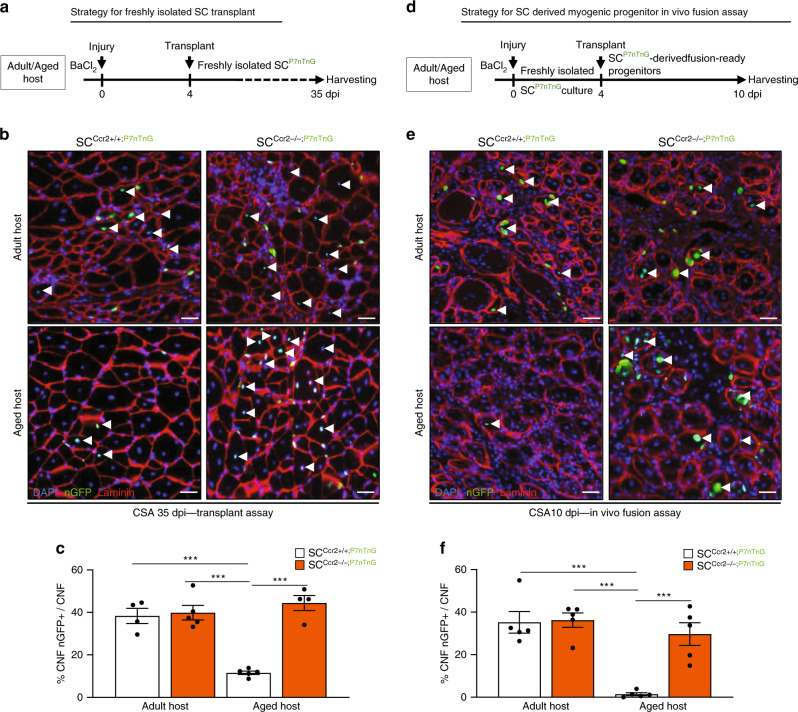
Elevated Ccr2 chemokines in aged regenerating muscle restrains MP contribution. **a** Experimental strategy for freshly isolated SC long-term transplant. Adult SCs derived from Ccr2^+/+^; Pax7^CreER/+^; Rosa26^nTnG/+^ (SC^Ccr2+/+; P7nTnG^; *n* = 4) or Ccr2^−/−^; Pax7^CreER/+^; Rosa26^nTnG/+^ (SC^Ccr2−/−; P7nTnG^; *n* = 4) are indelibly labelled for nuclear GFP (nGFP) allowing to track engraftment and contribution to regeneration. 3000 freshly isolated nGFP SCs were transplanted into an adult or aged regenerating muscle at 4dpi, and contribution analyzed at 35dpi. **b**, **c** Representative images (**b**) and **c** quantification of long-term transplanted adult (*n* = 5 mice per condition) and aged (*n* = 5 mice per condition) muscles. **d** Experimental strategy for freshly isolated SC culture to obtain fusion-ready MPs prior to transplant and in vivo fusion assays. Adult SCs derived from Ccr2^+/+^; Pax7^CreER/+^; Rosa26^nTnG/+^ (SC^Ccr2+/+; P7nTnG^; *n* = 4) or Ccr2^−/−^; Pax7^CreER/+^; Rosa26^nTnG/+^ (SC^Ccr2−/−; P7nTnG^; *n* = 4) were isolated and cultured on recombinant extra cellular matrix gels to obtain fusion-competent MPs. 15000 fusion-competent MPs were injected into adult or aged regenerating muscle (4dpi) that were analyzed at 10dpi for fusion competency. **e**, **f** Representative images (**e**) and **f** quantification of regenerating adult (*n* = 5 mice per condition) and aged (*n* = 5 mice per condition) muscles 6 days post-transplant (10dpi) for in vivo fusion assay. Successful engraftment was assessed by quantifying ratio of nGFP+ centrally nucleated fiber (CNF; regenerating) relative to total centrally nucleated fibers in the area of transplant. Area of transplant was determined by staining serial 10 μm cryosections through 2500 μm of the transplanted muscles. Analysis of the engraftment efficiency was confined to the area of transplant and acquired at 20X to keep the area consistent. Scale bars, 25 μm. *P < 0.05, **P < 0.01, ***P < 0.001, two-way ANOVA followed by Tukey’s multiple comparisons test.

Skeletal muscle regenerative capacity declines with age^22,33^. Early stages of aged muscle regeneration are characterized by delayed activation of SCs; however, similar SC and MP content is observed between adult (2-months-old mice) and aged (24-months-old mice) regenerating muscle at 5dpi (Supplementary Fig. 6a)^50^. Furthermore, assessment of SCs and MPs from aged 5dpi muscle demonstrated reduced *Myogenin* and increased *Ccr2* expression compared to adult (Supplementary Fig. 6b). To determine whether higher Ccr2 chemokines levels are a feature of aged muscle regeneration, we compared *Ccl2*, *Ccl7*, and *Ccl8* mRNA and protein expression between adult and aged 5dpi muscles. We found Ccr2-ligands were elevated in aged relative to adult 5dpi regenerating muscle (Fig. 5a and Supplementary Fig. 6c). We next asked whether healthy adult SC-derived progenitors could fuse into aged regenerating myofibers in an environment of high Ccr2 chemokines. Initially, we obtained freshly isolated SCs indelibly labeled with nuclear GFP (nGFP) from adult WT Pax7^CreER/+^; Rosa26^nTnG/+^; Ccr2^+/+^ (SC^Ccr2+/+;P7nTnG^) or Pax7^CreER/+^; Rosa26^nTnG/+^; Ccr2^−/−^ (SC^Ccr2−/−;P7nTnG^) mice, and subsequently transplanted 3,000 of them into regenerating adult and aged muscle hosts (Fig. 5b). Freshly isolated adult SC^Ccr2+/+;P7nTnG^ and SC^Ccr2−/−;P7nTnG^ displayed similar engraftment efficiency into adult regenerating hosts (Fig. 5c, d). Consistent with previous reports, freshly isolated adult SC^Ccr2+/+;P7nTnG^ displayed poor engraftment into an aged regenerating host (Fig. 5c, d)^51^. In contrast, freshly isolated SC^Ccr2−/−;P7nTnG^ engraftment into aged or adult regenerating hosts was similar (Fig. 5c, d). Similar results were obtained when total number of nGFP donor contribution in each treatment group was normalized to muscle volume (Supplementary Fig. 7a). In addition, we found that nGFP+ regenerated myofibers from SC^Ccr2−/−;P7nTnG^ recipients were significantly larger in size (Supplementary Fig. 7b). Thus, Ccr2 deficiency in freshly isolated adult SCs was sufficient to improve their engraftment and contribution into aged regenerating hosts.

Next, we wanted to determine if the improved engraftment of adult Ccr2^−/−;P7nTnG^ SC into aged regenerating hosts reflects enhanced fusion competency. To do so, we developed an in vivo MP fusion assay. To obtain *fusion-ready* MPs, we cultured freshly isolated adult SC^Ccr2+/+;P7nTnG^ or SC^Ccr2−/−;P7nTnG^ cells in growth media on recombinant extracellular matrix gel for 4 days. Under these conditions SC^Ccr2+/+;P7nTnG^ and SC^Ccr2−/−;P7nTnG^ display similar growth in culture (Fig. 2f and Supplementary Fig. 8a). Subsequently, 15,000 SC^Ccr2+/+;P7nTnG^ and SC^Ccr2−/−;P7nTnG^ derived MPs were intramuscularly injected into adult or aged regenerating 4dpi muscle hosts (Fig. 5d). Remaining cells were re-plated at the same density and displayed similar survival in culture (Supplementary Fig. 8b). Consistent with the propensity for MPs to rapidly proceed toward terminal commitment, SC^Ccr2+/+;P7nTnG^ and SC^Ccr2−/−;P7nTnG^ derived progenitor fusion was local to the site of intramuscular delivery and along the needle track, which was defined as the area of engraftment (Supplementary Fig. 8c). To quantify SC^Ccr2+/+;P7nTnG^ and SC^Ccr2−/−;P7nTnG^ derived MP fusion, we assessed the presence of central nGFP-positive myofibers. In adult regenerating myofiber hosts, both SC^Ccr2+/+;P7nTnG^ and SC^Ccr2−/−;P7nTnG^ derived MPs displayed similar fusion capacity (Fig. 5e, f and Supplementary Fig. 8d). However, SC^Ccr2+/+;P7nTnG^ derived progenitors displayed limited, if any, fusion into aged regenerating myofibers (Fig. 5e, f and Supplementary Fig. 8d). In contrast, fusion of SC^Ccr2−/−;P7nTnG^ derived MPs was similar regardless of regenerating host myofiber age (Fig. 5e, f and Supplementary Fig. 8d). We also found that nGFP+ aged regenerating myofibers in SC^Ccr2−/−;P7nTnG^ recipients displayed larger fiber size (Supplementary Fig. 8e). Thus, *Ccr2* deficiency, specifically in SC derived MPs, is sufficient to overcome the inhibitory effects of the aged regenerating environment enriched in Ccr2 chemokines; thereby, promoting fusion of MPs and their contribution to aged muscle regeneration.

### Timely inhibition of Ccr2 promotes aged muscle recovery

**Fig. 6:**
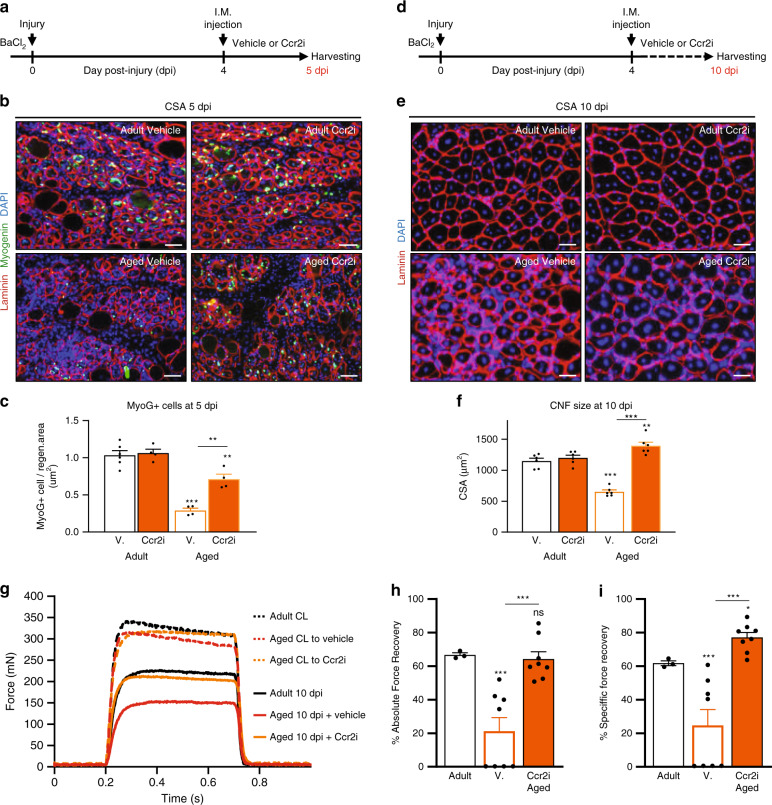
Timely inhibition of Ccr2 promotes aged muscle regeneration and strength recovery. **a** Schematic for acute muscle injury followed by single intramuscular injection of Ccr2 inhibitor (Ccr2i) or Saline (Vehicle) at 4 days post injury (4dpi). At 5dpi muscles were collected to assess MyoG+ MPs. **b**, **c** Immunofluorescence of MyoG+ MPs in adult (4 months old; *n* = 6 for vehicle and *n* = 6 for Ccr2i) and aged (24 months old; *n* = 4 per condition) 5dpi muscles (**b**) and **c** number of MyoG+ cells per area of regeneration (regen. area; um^2^). Laminin (Red); MyoG (Green); DAPI (Blue). **d** Schematic depicting the strategy to assess cross sectional area (CSA; um^2^) of regenerated fibers (centrally nucleated fibers; CNF) in adult and aged 10dpi muscles that received a single intramuscular injection of Ccr2i or Vehicle at 4dpi. **e**, **f** Representative images (**e**) and **f** quantification of regenerated fibers CSA in adult (*n* = 5 per condition) and aged (*n* = 5 per condition) mice at 10dpi. Laminin (Red); DAPI (Blue). **g**, Representative force curves from 10dpi and uninjured, non-injected contralateral (CL) EDL muscles from adult or aged mice treated with Vehicle or Ccr2i at 4dpi. **h**, **i** Quantification of the **h** absolute force recovery and **i** specific force recovery in regenerated muscles from adult (*n* = 3 mice) and aged (*n* = 8 mice per condition). Data are reported as mean ± s.e.m. Scale bars, **b** 100 μm, **e** 25 μm. non-significant (n.s.) *P* > 0.05; *P < 0.05, **P < 0.01, ***P < 0.001, non-parametric one-way or two-way ANOVA followed by Tukey’s multiple comparisons test.

Next, we sought to determine the consequences of the timely direct delivery of Ccr2i on adult and aged muscle regeneration. To this end, we intramuscularly injected vehicle or Ccr2i to adult or aged regenerating muscle at 4dpi (Fig. 6a–f). Subsequently, we examined fusion competent MyoG+ MP number at 5dpi (Fig. 6a, b), and regenerated myofiber size at 10dpi (Fig. 6d, e). Consistent with low levels of Ccr2 chemokines, we found no effect of Ccr2i direct delivery on adult muscle regeneration in terms of MyoG+ cell number at 5dpi or regenerated myofiber size at 10dpi (Fig. 6a–f). In contrast, direct intramuscular delivery of Ccr2i to regenerating aged muscle at 4dpi resulted in increased MyoG+ cell number at 5dpi, and increased regenerated myofiber size at 10dpi (Fig. 6a–f). To determine if Ccr2i treatment can directly influence MP fate in an in vivo context, fusion-ready SC^Ccr2+/+;P7nTnG^ MPs were pre-treated with Ccr2i, washed, and transplanted into an aged regenerating host (Supplementary Fig. 9a). Pre-treatment of SC^Ccr2+/+;P7nTnG^ MPs with Ccr2i led to their improved fusion into aged regenerating myofibers and larger nGFP+ myofibers (Supplementary Fig. 9b–e). Although adult SCs are sensitive to Ccr2 chemokines, whether aged SCs retain this property is unknown. Similar to adult, aged SC and derived MPs exposed to Ccl^2/7/8^ in culture displayed no change in MyoD+ proportions (Supplementary Fig. 10a, b) and had reduced myotube formation, in a Ccr2-dependent fashion (Supplementary Fig. 10c, d).

Finally, as a fundamental test of functional recovery, we assessed if direct Ccr2i delivery improved muscle strength of aged muscle post injury. To this end, we examined intrinsic absolute and specific force recovery of regenerating EDL muscles at 10dpi relative to contralateral (CL) uninjured muscles, after intramuscular injection of Ccr2i or vehicle at 4dpi^30,52–54^ (Fig. 6d and Supplementary Fig. 11a). As expected, injured muscles produced less force than their uninjured counterparts regardless of age (Fig. 6g and Supplementary Fig. 11b, c). However, in comparison to adult the extent of aged 10dpi muscle force recovery relative to uninjured contralateral controls was reduced (20% vs 60%) (Fig. 6g–i and Supplementary Fig. 11b, c). Furthermore, half of vehicle treated aged regenerating muscles at 10dpi were composed primarily of connective tissue (* in left picture) and unable to elicit recognizable force values in response to direct ex vivo muscle stimulation (Fig. 6g–i and Supplementary Fig. 11a–c). Strikingly, all Ccr2i treated aged regenerating muscles at 10dpi were able to generate force. In addition, Ccr2i treatment promoted force recovery in injured aged muscle (Fig. 6h, i and Supplementary Fig. 11b, c). Collectively, our findings demonstrate that timely pharmacological inhibition of Ccr2 is a viable strategy to promote both the regeneration and functional recovery of aged muscles after a traumatic injury.

## Discussion

In response to injury, the coordinated activation, proliferation, differentiation and fusion of SC derived MPs is required for skeletal muscle regeneration. The complex aged inflammatory environment disrupts this synchronization^33,40^. The CC chemokine receptor Ccr2 is a regulator of immune cell recruitment and function during muscle regeneration; however, a role of Ccr2 in non-hematopoietic cells in the context of tissue regeneration and aging is unclear. In this study, we demonstrate that active SCs and MPs in regenerating muscle express Ccr2. Although dispensable for MP expansion and growth, elevated Ccr2 chemokine activity inhibits MP fusion and contribution to multinucleated myofiber formation. After an acute injury, aged muscle is characterized by high Ccr2 chemokines levels. We demonstrated that *Ccr2* deficiency or timely Ccr2 inhibition promotes MP fusion and aged skeletal muscle regeneration, respectively.

Although one study employed antibody labeling to detect Ccr2 in MPs of regenerating muscle^23^, the use of Ccr2 reporter mice has been the gold standard method to evaluate *Ccr2* expression, due to the elevated turnover of G protein-coupled receptors^26,36^. We found SCs from uninjured muscle express negligible levels of *Ccr2* reporter. In contrast, we demonstrate, using multiple methods, that active SCs and MPs from regenerating skeletal muscle express relatively higher levels of *Ccr2*. Accordingly, RNA-seq analysis of SCs and MPs from uninjured and regenerating muscle demonstrate similar patterns of *Ccr2* expression as observed here^55^. Furthermore, we observe that *Ccr2* expression in active SCs and MPs is positively associated with Ccr2 chemokines levels, suggesting *Ccr2* expression can be enhanced by its own ligands.

We find that ectopic treatment with Ccr2 chemokines inhibited the capacity of adult SCs and derived MPs to contribute to multinucleated myofiber formation. A previous report demonstrated alterations in proliferation are not observed in adult Ccr2^−/−^ MP cells; however, neither Ccr2^+/+^ or Ccr2^−/−^ MPs were treated with Ccr2 chemokines^39^. Although we find Ccr2 deficiency did not alter SC growth in standard culture conditions, we did find Ccr2^−/−^ SC-derived MPs were resistant to Ccr2 chemokines-mediated inhibition of terminal differentiation and fusion. Previous groups have demonstrated roles for p38MAPK signaling in regulating SC fate and MP function^42–44^. The p38MAPK family is composed of four genes *Mapk14, Mapk11*, *Mapk12*, and *Mapk13*, encoding for the kinases p38α, p38β, p38γ and p38δ, respectively. Activation of p38α/β, often referred to as p38MAPK, induces myogenic differentiation^42^, whereas activation of p38δ/γ inhibits myogenic differentiation^43,44^. Consistent with the latter, we found that Ccr2-mediated inhibition of MP fusion and myotube formation is associated with activation of p38δ/γ^43,44^. Considering the recent discovery of proteins with critical roles in MP fusion^56–58^; it will be of interest to determine if Ccr2 and p38δ/γ activity can modulate these fusion regulators.

During adult muscle regeneration, high levels of Ccr2-ligands coincide with the recruitment of inflammatory cells and SC expansion. Later stages of adult muscle regeneration are associated with the resolution of inflammation, progression toward terminal myogenic differentiation and decreased levels of Ccr2-ligands in the environment^59^. Following an acute injury, we uncovered the prolonged elevated levels of Ccr2 chemokines in aged regenerating muscles. This was consistent with a decrease in the numbers of fusion competent myogenic cells in 5dpi regenerating aged in comparison to adult muscles in vivo. Indeed, *Ccr2* deficiency promoted SC-derived MP fusion to aged regenerating myofibers. Furthermore, timely Ccr2 inhibition stimulated aged muscle regeneration and functional recovery after an acute degenerative injury. Ccr2-signaling acts at the nexus of events regulating muscle regeneration in what is likely a complex interplay between immune, and myogenic cells^1^. Therefore, timely Ccr2i treatment could alter the levels of inflammatory derived factors that influence MP fate^60^. A subset of aged SCs retain extensive regenerative potential, thus, timely Ccr2 treatment may primarily alter the response of relatively healthy aged SCs and derived MPs to high Ccr2 chemokines in the aged environment^31,32^. Alternatively, opportune Ccr2i treatment could restore the function of some SCs prone to senescence^22,50^. Indeed, Ccr2 signaling is part of the senescence associated secretory pathway, and can reinforce senescence programs^3,61,62^. Therefore, it is conceivable that transient Ccr2i treatment could attenuate senescence-like phenotypes induced by age-related dysregulation in protein and mitochondrial quality control in some SCs and derived MPs^22,63^.

Although aged human skeletal muscle can regenerate^40^, delays in this process cause persistent physical discomfort that compromises recovery, mobility, and independence in the growing elderly population^64–66^. Here, we identify a previously uncharacterized role for Ccr2 signaling as an inhibitor of aged muscle regeneration. Furthermore, we demonstrate targeting Ccr2 can promote MP function in an aged environment, and thereby restore aged muscle function after a degenerative injury. In light of our observations, the identification of similar modes of stem cell and progenitor fate regulation in other systems could prove useful to improve aged tissue regeneration. Specifically, timely inhibition of Ccr2 could be used to rejuvenate aged tissue regeneration or improve cell-based therapies in a high inflammatory environment. Finally, the recent clinical trials using Ccr2 inhibitors in diverse human disorders suggest targeting Ccr2 is a viable treatment^16–19,67^. These studies could give us further insight into the systemic effects of Ccr2 inhibition and help to develop future therapeutic strategies for aged tissue regeneration in a clinically relevant context.

## Methods

### Animal studies

This study was carried out in strict accordance with the recommendations in the Guide for the Care and Use of Laboratory Animals of the National Institutes of Health. All procedures and protocols involving animals were approved and authorized by the Institutional Animal Care and Use Committee (IACUC) at the University of Rochester called the University Committee on Animal Resources (UCAR). C57BL6 adult (2–4 months; Jackson Labs) and aged (24–25 months; National Institute on Aging) mice were used for indicated experiments. *Ccr2-KO* (Ccr2^−/−^) (004999) and *Ccr2-KI/KO* (Ccr2^GFP/GFP^) (027619) mice were obtained from Jackson Labs. For transplant experiments, we used *Pax7*^*CreER/+;*^ *Rosa26*^*nTnG/+*^ mice^30^. All mice were housed in the Vivarium animal housing areas at URMC and were cared for by the facility staff following the programs for animal care set by the University of Rochester. The Animal Resource program follows state and federal laws, NIH policies, and is accredited by the Association for Assessment and Accreditation of Laboratory Animal Care International.

### Skeletal muscle injury

Mice were anesthetized by 3% isoflurane inhalation. Buprenorphine (0.1 mg/kg) was administered prior to the procedure and approximately every 12 h as needed. The skin overlaying the tibialis anterior (TA) muscle was shaved and the TA and extensor digitorum longus (EDL) were directly injected with a 1.2% solution of BaCl_2_ in normal saline.

### Transplant and in vivo fusion assay

For freshly isolated SC transplant, cells were derived from uninjured limb from adult *Pax7*^*CreER/+;*^ *Rosa26*^*nTnG/+*^ mice wildtype for Ccr2 (SC^Ccr2+/+^) or mutant (SC^Ccr2−/−^). Cells were counted and 3000 SCs were transplanted into a pre-injured TA/EDL of an adult or aged mouse host (4dpi). 35 days post injury, transplanted muscles were collected, frozen, sectioned and stained for GFP, Laminin and DAPI. Engraftment and contribution to regeneration was assessed by quantifying number of centrally nucleated fiber with GFP+ nuclei within the area of engraftment.

For in vivo fusion assay, the experiment was similar, except we cultured cells for 96 h prior to transplant. Cell growth was controlled throughout by quantifying number of live nGFP cells in culture (Supplementary Fig. 8a), while survival post-trypsinization was verified by re-plating extra cells (Supplementary Fig. 8b). For in vivo fusion assays, 15000 cells were injected into an injured limb (4dpi). Area of engraftment was determined within the field of view containing centrally nucleated nGFP+ fibers, as depicted in Supplementary Fig. 10c.

### Physiologic muscle force generation assay

Muscle force generation capacity was analyzed in EDL muscles using an ASI muscle contraction system (Aurora Scientific)^30^. Mice were maintained under isoflurane throughout the procedure. TA was removed, and EDL were dissected, adjusted to optimal length (OL), and tested at different frequencies to determine absolute force values. Muscle force was recorded and analyzed using Dynamic Muscle Control and GraphPad Prism software. Physiologic cross-sectional area was calculated using the equation of cross-sectional area = muscle mass/[muscle density (1.06 g/cm^3^) × optimal fiber length (0.44 × OL)]. Force of recovery was calculated based on the injured normalized to contralateral uninjured force values at 150 Hz stimulation.

### Cell isolation and culture

For magnetic activated cell sorting (MACS), skeletal muscles were dissociated in F10+ media (F10 supplemented with 10% horse serum, and 1% HEPES) containing 0.2% Collagenase II and 0.4% Dispase using the Gentle MACS tissue dissociator for 30–60 min at 37 °C. Single cell suspension was spun down at 500 g for 20 min at 4 °C, washed with F10+ media and filtered using Smart Filters (Miltenyi Biotec). Cells were resuspended in ice-cold PBS 0.5% BSA prior to filtration through a FACS tube cap. SCs were then freshly isolated using first the Satellite Cell Isolation Kit (Miltenyi Biotec) for negative selection, followed by positive selection using α7Integrin beads following the manufacturer’s protocols.

SC cultures and related experiments were performed as previously described^34^. Briefly, 5000 SCs (72 h cultures) or 10000 SCs (differentiation assay) were plated on 0.5% ECM in growing media (GM; DMEM, 10%horse serum, FGF2, 1% pen/strep, 1% HEPES). GM was changed every 48 h and differentiation media (DM, DMEM, 2% horse serum, 1% pen/strp, 1% HEPES) was added at 96 h for 18–24 h, monitoring the formation of long and healthy myotubes. All cells were incubated at 37 °C in 5% CO_2_ and humid air conditions.

### FACS and flow cytometry analysis

For flow cytometry analysis, skeletal muscles were dissociated using the Gentle MACS (see above), and SCs were isolated as previously described^34^. Briefly, SCs were characterized as Cd45−, Sca1−, Cd31− (Lineage negative; Lin−) (BD Biosciences; Biolegend), and VCAM + and β1Integrin + (Biolegend). Muscle-derived monocytes (Cd11b ultrapure beads; Miltenyi) were used as controls for the Ccr2-GFP mouse experiments as they express high levels of Ccr2 in response to injury. Hematopoietic cells were selected for Cd45+ (BioLegend). Gating strategy is described in Supplementary Supplementary Fig. 1a and depict cell populations from injured muscles while representative FMO and single stain controls for the antibodies used in flow experiments can be found in Supplementary Fig. 12. Flow cytometry experiments were always performed with single stain, FMO and appropriate experimental controls. For cell sorting purposes, we used BD FACSDiva software v8, while flow analysis was performed on FCS Express v6.

### Transfection and siRNA treatments

Cells were transfected with siRNAs (Dharmacon) using Lipofectamine RNAi MAX (Invitrogen) according to the manufacturer’s instructions. siGLO was used as control. We used siRNA targeting other p38 genes as a control of specificity. None of the other p38 genes were downregulated when using siRNA targeting a specific p38 gene member (Supplementary Fig. 5e).

### Recombinant chemokines and Ccr2 inhibitor treatments

In culture, recombinant chemokines were delivered in PBS at a final concentration of 0.5–50 ng/mL as indicated, while BMS CCR2 22 inhibitor (Ccr2i) was used at 1 nM as previously described^68,69^. For in vivo experiments, Ccr2i was intramuscularly delivered in 90%Saline-10%EtOH at 0.5 ng/uL, as previously described^68^. Recombinant chemokines were delivered in PBS at a final concentration of 5 ng/mg body weight.

### Tissue section and immunostaining

For injury or transplant studies, TA muscles were harvested and frozen in isopentane cooled in liquid nitrogen and stored at −80 °C prior to sectioning or prepared for magnetic activated cell sorting (MACS). Frozen muscles were sectioned at 10 μm. For Hematoxilin and Eosin staining, sections were treated as previously described^30,34^. Muscle sections were fixed for 3 min in 4% paraformaldehyde (PFA), permeabilized with PBS-T (0.2% Triton X-100) for 10 min and blocked in 10% Normal Goat Serum (NGS; Jackson Immuno Research, West Grove, PA) in PBS-T for 30 min at room temperature. When mouse primary antibodies were used, sections were additionally blocked in 3% AffiniPure Fab fragment goat anti-mouse IgG(H + L) (Jackson Immuno Research) with 2% NGS in PBS at room temperature for 1 h. Primary antibody incubation in 10% NGS/PBS-T was carried out at 4 °C overnight or 2 hr at RT and sections were incubated with secondary antibodies in 10% NGS/PBS-T for 1 h at RT. DAPI staining was used to label nuclei. All slides were mounted with Fluoromount-G (SouthernBiotech, Birmingham, AL). At least three sections from three slides were analyzed per sample. Sections and cells were imaged on a Zeiss Axio Observer A.1 microscope (Germany) or Echo Revolve and processed and analyzed in ImageJ. Sample analysis was conducted in a double-blind manner. For transplant analysis, each transplanted muscle was sectioned at 10 μm through ~2500 μm. Area of transplant was determined by staining >20 muscle sections throughout the muscle for each sample and was then contained in a 20X field of view for analysis of nGFP+ centrally nucleated fibers. Analysis was performed using at least 60 fields of view.

### Immunoblotting and Luminex

Cell lysates (50 mM HEPES [pH 7.4], 150 mM NaCl, and 1% Triton X-100) and freshly added EDTA-free protease and phosphatase inhibitor cocktails (Roche) were immunoblotted with primary antibodies overnight at 4 °C. HRP-conjugated secondaries and ECL from BioRAD was used for chemiluminescence detection, while Dylight secondary antibodies were used for infrared detection on LI-COR Odyssey and using LI-COR Image Studio software or Image Studio Light for analysis and band quantification. Fluorescent images were saved in grey scale and therefore displayed as such. Uncropped and unprocessed blot images with molecular weight are available in the Source Data File.

For Luminex assays, samples were prepared using a customized Mouse Magnetic Luminex Assay (R&D Systems) following manufacturer’s protocol and using BioRad BioPlex 200 apparatus and software.

### RNA extraction and qPCR

RNA was extracted using TRIzol reagent (Life Technology) following the manufacturer’s protocol. RNA was quantified, normalized, and used for RT-PCR followed by qPCR using EvaGreen (Bio-Rad) and the 7500 Fast Real-Time PCR system (Applied Biosystems) with StepOnePlus software to collect and analyze data. Transcript levels were normalized to an average of *GAPDH* and *B2M* and then to the control condition.

For qPCR, we used the following primers:

MyoG_Forward Primer 5′-GTCCCAACCCAGGAGATCAT-3′

MyoG_Reverse Primer 5′-CCACGATGGACGTAAGGGAG-3′

Ccr2_Forward Primer 5′-AGGAGCCATACCTGTAAATGC-3′

Ccr2_Reverse Primer 5′-TGTGGTGAATCCAATGCCCT-3′

Mapk14_Forward Primer 5′-TGACCCTTATGACCAGTCCTTT-3′

Mapk14_Reverse Primer 5′-GTCAGGCTCTTCCACTCATCTAT-3′

Mapk11_Forward Primer 5′-GCGGGATTCTACCGGCAAG-3′

Mapk11_Reverse Primer 5′-GAGCAGACTGAGCCGTAGG-3′

Mapk13_Forward Primer 5′-ATGAGCCTCACTCGGAAAAGG-3′

Mapk13_Reverse Primer 5′-GCATGTGCTTCAAGAGCAGAA-3′

Mapk12_Forward Primer 5′-AAGGGCTTTTACCGCCAGG-3′

Mapk12_Reverse Primer 5′-GGCGCAACTCTCTGTAGGC-3′

### Antibodies

The following antibodies were used: mouse anti-Pax7 (1:100, Developmental Studies Hybridoma Bank (DSHB), Iowa City, IA), mouse anti-MyoD (BD Biosciences #554130), mouse anti-phospho-MyoD (1:1000, Sigma) rabbit anti-Myogenin (1:250, AbCam), rat or rabbit anti-Laminin (1:1000 or 1:1500, Sigma-Aldrich, L0663 or L9393), rabbit anti-skeletal muscle myosin (1:250, Sigma-Aldrich HPA1239), rabbit anti-Ccr2 (1:500, AbCam), rabbit or mouse anti-p38 (1:1000, Cell Signaling), rabbit or mouse anti-phospho-p38 (1:1000, Cell Signaling), rabbit anti-phospho-p38delta/gamma (1:1000, ThermoFisher), rabbit anti-Erk1/2 (1:1000, Cell Signaling), mouse anti-phospho-Erk1/2 (1:1000, Cell Signaling). All antibodies were previously confirmed for specificity in the literature and/or by the manufacturers.

### Statistical analysis

Statistical significance was assessed using Prism8 software via Student’s or Welch’s *t*-test (unpaired, two-sided, 95% confidence interval [CI]) or ANOVA (one way or two way, followed by Tukey post hoc test; 95% CI). Error bars are reported as s.e.m. or s.d. and displayed in appropriate graphs. *p* values < 0.05 were considered statistically significant (^∗^*p* < 0.05, ^∗∗^*p* < 0.01, and ^∗∗∗^*p* < 0.001). No power analysis was performed. Sample size was based on previous experiments.

### Reporting summary

Further information on research design is available in the Nature Research Reporting Summary linked to this article.

## Supplementary information

Supplementary Information

Reporting Summary

## Data Availability

All the data supporting the findings of this study are available from the corresponding author upon reasonable request. Source data are provided with this paper.

## Source data

Source Data
