# Supplementary material for: Inhibition of inflammatory CCR2 signaling promotes aged muscle regeneration and strength recovery after injury
Source: Nat Commun. 2020 Aug 20;11:4167. doi: 10.1038/s41467-020-17620-8 (PMC7441393; doi:10.1038/s41467-020-17620-8)
Supplement: Supplementary file 1 — Supplementary Information [file 41467_2020_17620_MOESM1_ESM.pdf]

Title: Inhibition of inflammatory CCR2 signaling promotes aged muscle regeneration and strength recovery after injury

Blanc S. Roméo et al., 2020.

Supplementary Figure 1

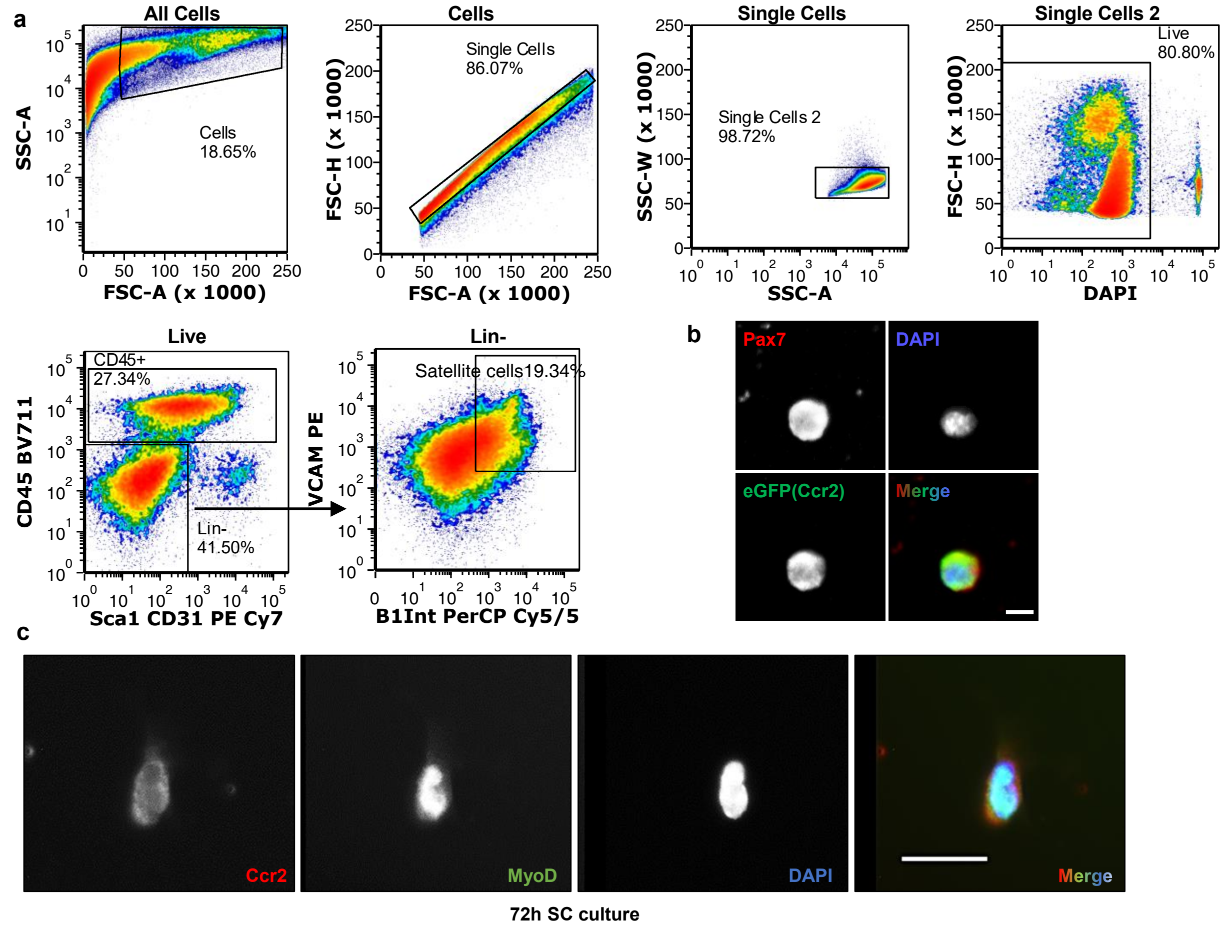

Supplementary Figure 1: Strategy for analysis of Ccr2-expressing cell populations during adult skeletal muscle regeneration

a, Muscles were harvested and dissociated prior to flow-cytometry analysis. Cell suspension was gated for DAPI (live/dead) and discriminated for doublets prior to analysis of hematopoietic cells (CD45+) and satellite cells (SCs) using a combination of cell surface markers. CD45/CD31/Sca1 was used as lineage negative markers (Lin-) to identify VCAM+ β1-Integrin+ SCs. **b,** Representative immunohistochemistry image of a Pax7+ eGFP+(encoded under the *Ccr2* gene) satellite cells (SCs) isolated by FACS and plated for 12 hours prior to fixation and staining. Scale bars, (a) 10µm, (b) 25µm. (n = 1 experiment) **c,** Immunostaining of MyoD+ myoblast derived from freshly isolated SCs cultured for 72h showing Ccr2 expression at the membrane (n = 1 experiment).

Supplementary Figure 2

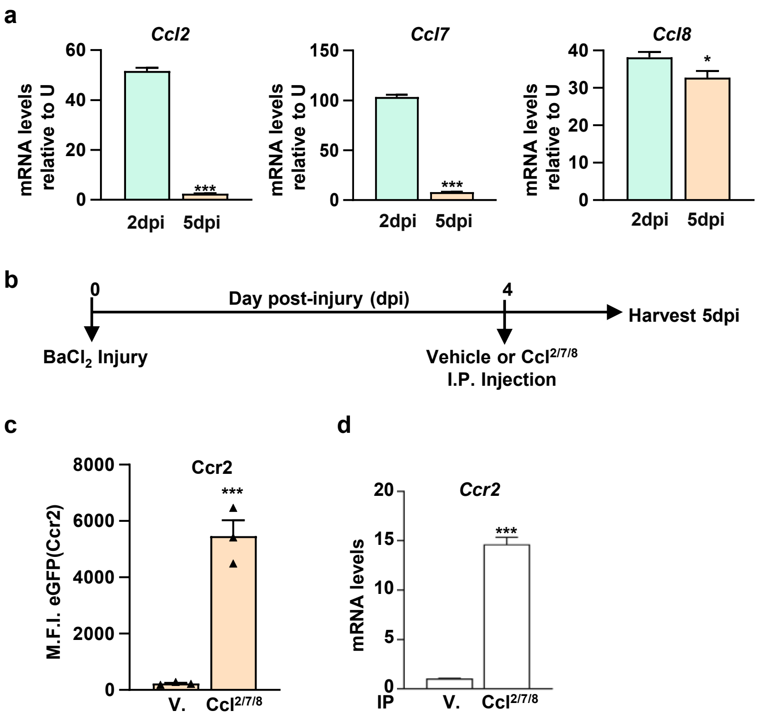

Supplementary Figure 2: Analysis of Ccr2 chemokines levels during adult skeletal muscle regeneration

a, Ccr2 ligands (Ccl2, Ccl7 and Ccl8) levels of mRNA in adult muscles following injury. Experiments were performed using adult uninjured and injured tibialis anterior (TA) muscles, 2- and 5-days post injury (2dpi and 5dpi). **b,** Strategy to systemically extend Ccr2 ligands ($Ccl^{2/7/8}$) in injured mice. Adult mice (2 months old) were injured with intramuscular (IM) injection of barium chloride, followed by intraperitoneal (IP) injection of recombinant chemokines or saline (vehicle) at 4dpi and harvested for analysis at 24 hours post-injection (5dpi). **c, d,** Expression of Ccr2 in SCs derived from adult injured muscles at 5dpi treated with vehicle (V) or $Ccl^{2/7/8}$ depicted by **(c)** eGFP mean fluorescence intensity (MFI) from flow cytometry analysis and **(d)** qPCR analysis. mRNA levels are reported as fold-change ± s.d. relative to *Gapdh* and *B2m* and normalized to uninjured. MFI is reported as mean±s.e.m. For each condition $n = 3$ mice. $^{*}P < 0.05$, $^{**}P < 0.01$, $^{***}P < 0.001$, unpaired two-tailed Welch's t-test.

Supplementary Figure 3

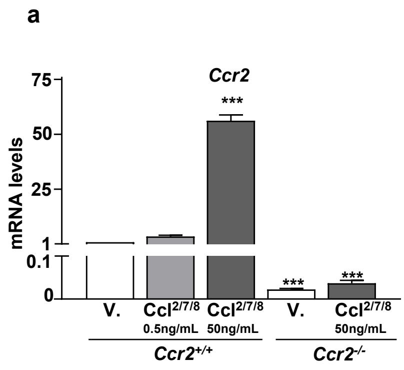

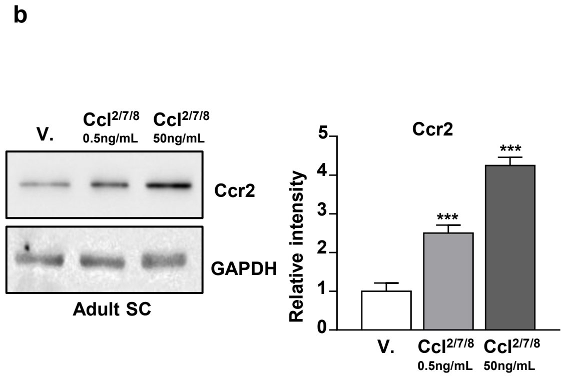

Supplementary Figure 3: Ccr2 levels in SC derived myogenic progenitors is regulated by levels of its chemokine ligands in a dose dependent manner

a, Rt-qPCR analysis from wildtype and $Ccr2^{-/-}$ adult SCs cultured with low (0.5ng/mL) or high dose (50ng/mL) of Ccr2 ligands shows dose-dependent increase in Ccr2 expression. **b, c, (b)** Immunoblotting from adult SCs cultured with low (0.5ng/mL) or high dose (50ng/mL) of Ccr2-ligands and **(c)** quantification show elevation of Ccr2 protein levels in dose-dependent fashion. GAPDH was used as loading control. For each condition $n = 3$ mice. Data are mean ± s.e.m. n.s. not significant, *$P < 0.05$, **$P < 0.01$, ***$P < 0.001$, non-parametric one-way ANOVA followed by Tukey's multiple comparisons test.

Supplementary Figure 4

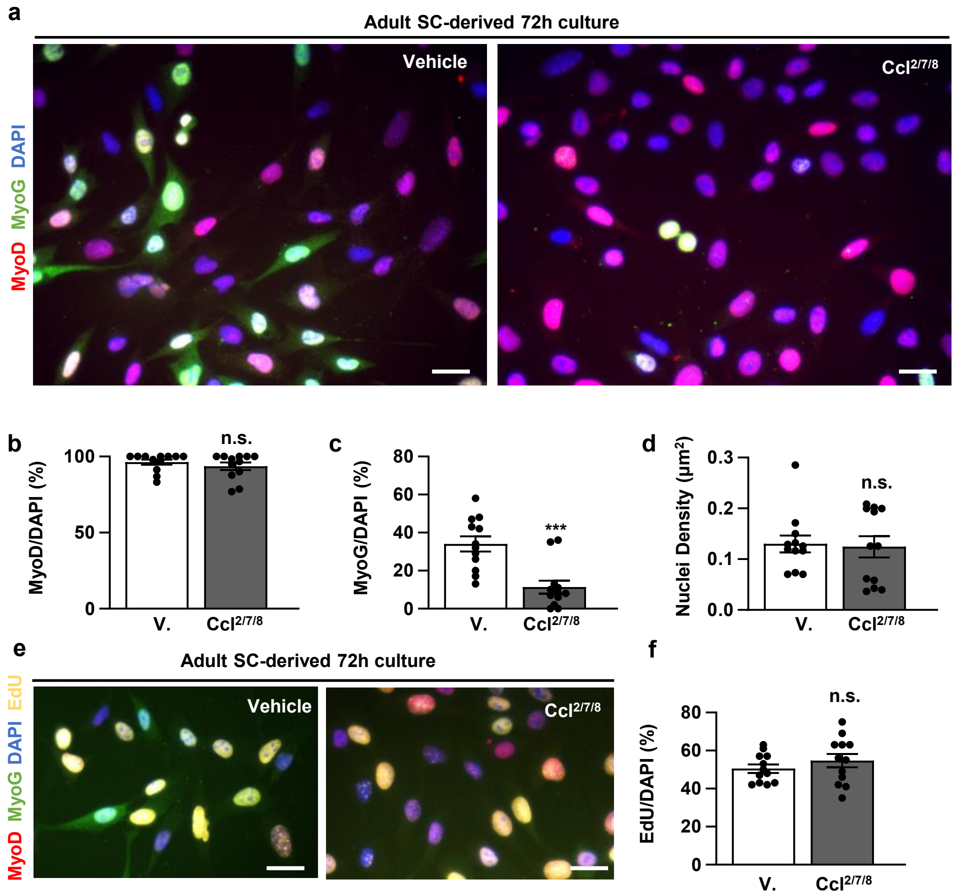

Supplementary Figure 4: Ccr2 activity in satellite cell-derived myogenic progenitors inhibit myogenic differentiation

a, Representative pictures of freshly isolated adult SCs cultured for 72 hours and treated with saline (Vehicle) or recombinant Ccr2-ligands ($Ccl^{2/7/8}$) 24 hours prior fixation. **b, c** Percentage of **(c)** myogenic progenitors (MyoD+) and **(d)** terminally committed myogenic progenitors (MyoG+) derived from adult SC-derived after 72 hours culture. **e, f,** Representative images (e) and quantification (f) of 72h SC-derived cultures to assess proliferation (EdU+ cells) of myogenic cells (MyoD+, MyoG+). Cells were pulsed with EdU 4 hours prior to harvest. Percentages are relative to total number of DAPI+ nuclei in field of view. Data are mean ± s.e.m. For each experiment, n = 12 mice per group from at least three experiments, >300cells, at least two wells per condition. Scale bars, 20µm. n.s. not significant, *P < 0.05, **P < 0.01, ***P < 0.001, unpaired two-tailed Welch's t-test.

Supplementary Figure 5

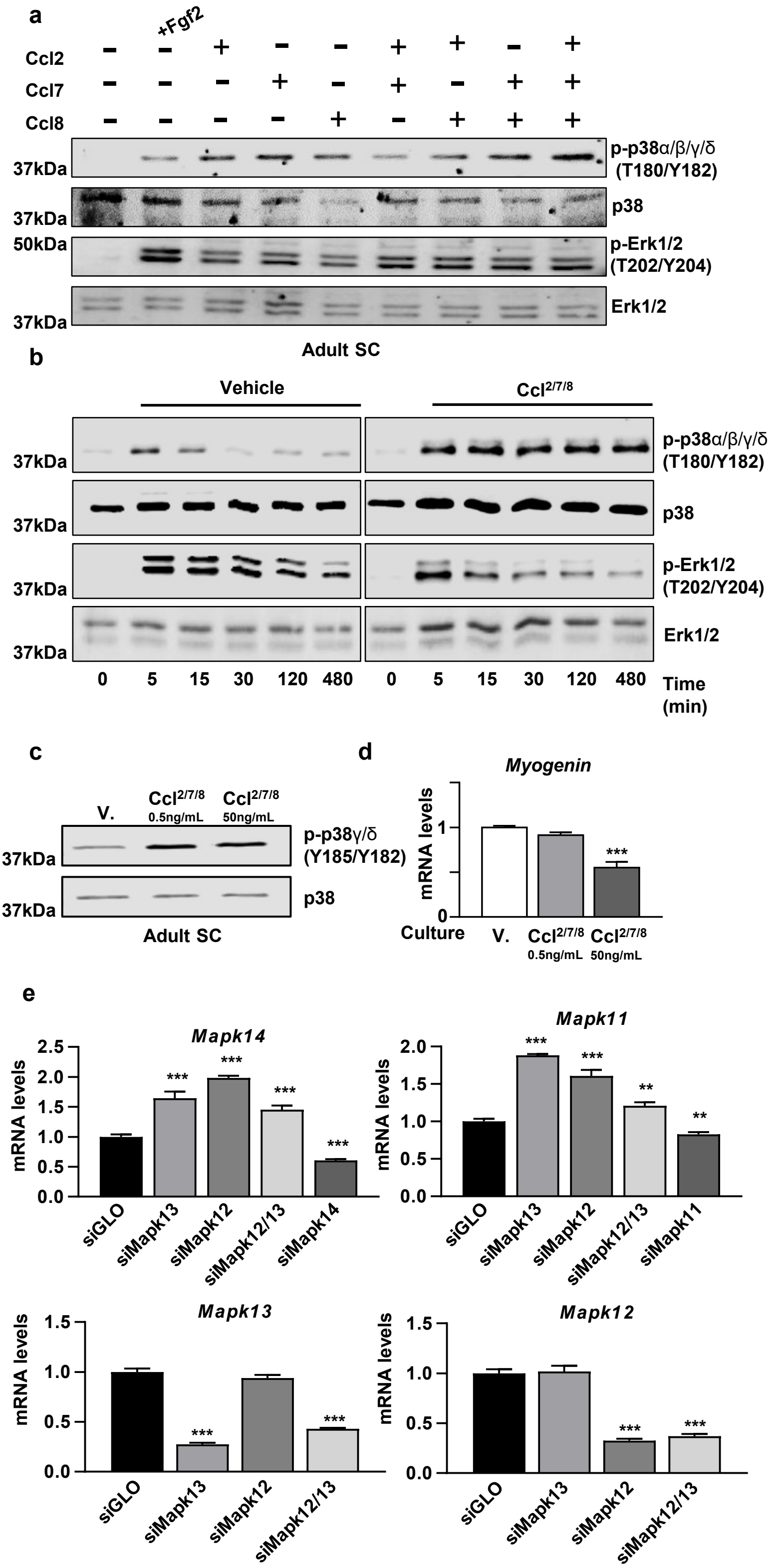

Supplementary Figure 5: Ccr2 activity in myogenic progenitors inhibits *Myogenin* and sustainably activates p38MAPK in a dose-dependent manner

a, Immunoblotting showing MAPK signaling activation in response to combinations of 30 minutes treatments on freshly isolated adult SCs cultured for 72 hours. Combination of Ccl2, Ccl7 and Ccl8 ($Ccl^{2/7/8}$; far-right lane) is required to simultaneously induce elevated phospho-p38MAPK and phospho-ErkMAPK. n = 3 mice. **b,** Kinetic of signaling activation in freshly isolated adult SCs cultured for 72 hours in response to $Ccl^{2/7/8}$. Immunoblots show sustained activation of p38MAPK. n = 3 mice. **c, d, (c)** Immunoblotting of p38MAPK signaling members p38γ and p38δ from adult SCs cultured for 96 hours in growth media followed by 24h in low serum media supplemented with low (0.5ng/mL) or high dose (50ng/mL) and **(d)** qPCR analysis show elevation of p38γ/δ and repression of *myogenin* in dose-dependent fashion (n = 3 mice). **e,** Transcript levels of p38MAPK gene members following single or combination treatment of siRNA. siGLO was used as control. (n = 3 experiments). Data are mean ± s.e.m. n.s. not significant, $^{*}P < 0.05$, $^{**}P < 0.01$, $^{***}P < 0.001$, non-parametric one-way ANOVA followed by Tukey's multiple comparisons test.

Supplementary Figure 6

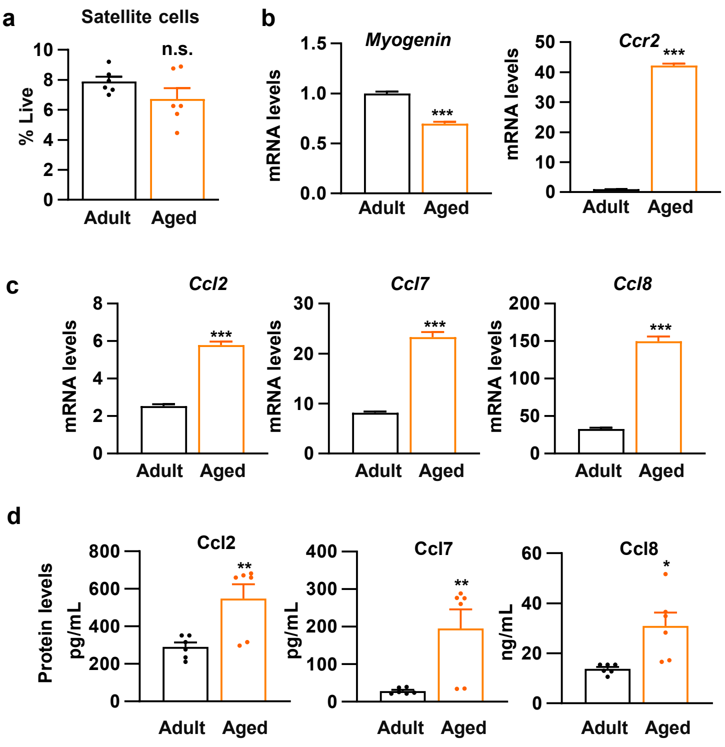

Supplementary Figure 6: Aged regenerating muscles display a myogenic inhibitory environment high in Ccr2 chemokines

a, Percentage of live satellite cells (Lin-Vcam+β1Int+) derived from adult (4 months old; n = 5) and aged (24 months old; n = 5) mice injured tibialis anterior (TA) muscle 5 days post injury (5dpi). Data are mean ± s.e.m. **b,** Transcript levels of *Myogenin* and *Ccr2* in SCs and myogenic progenitors isolated from 5dpi adult (n = 3) and aged (n = 3) muscle. **c,** mRNA levels of Ccr2-chemokine ligands in adult (4 months old; n = 6) and aged (24 months old; n = 6) regenerating muscles (5dpi). (b, c) mRNA levels are reported as fold-change ± s.d. relative to *Gapdh* and *B2m* and normalized to adult. **d,** Quantification of Ccr2-ligand chemokines (Ccl2, Ccl7 and Ccl8) concentration in adult (4 months old; n = 6) and aged (24 months old; n = 6) mice regenerating muscles 5 days post injury (5dpi). Data are reported as mean ± s.e.m. n.s. not significant, *$P < 0.05$, **$P < 0.01$, ***$P < 0.001$, unpaired two-tailed Welch's t-test.

Supplementary Figure 7

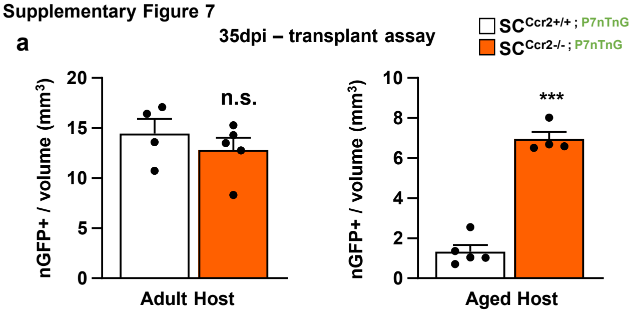

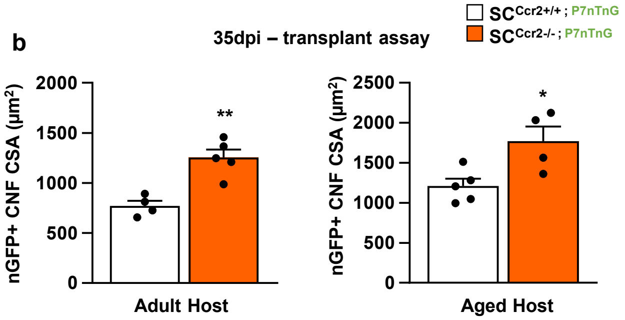

Supplementary Figure 7: Assessment of satellite cell transplant efficiency and effects on host muscle fibers regeneration

a, Quantification of absolute fused GFP+ nuclei normalized to host muscle volume (mm3) to assess transplant efficiency (n = 4-5 mice per experimental condition). **b,** Quantification of nGFP+ centrally nucleated fibers (CNF) size (μm2) to assess contribution of fused GFP+ cells to regeneration. (n = 4-5 mice per experimental condition). Data are mean ± s.e.m. P < 0.05, **P < 0.01, ***P < 0.001, unpaired two-tailed Welch's t-test.

Supplementary Figure 8

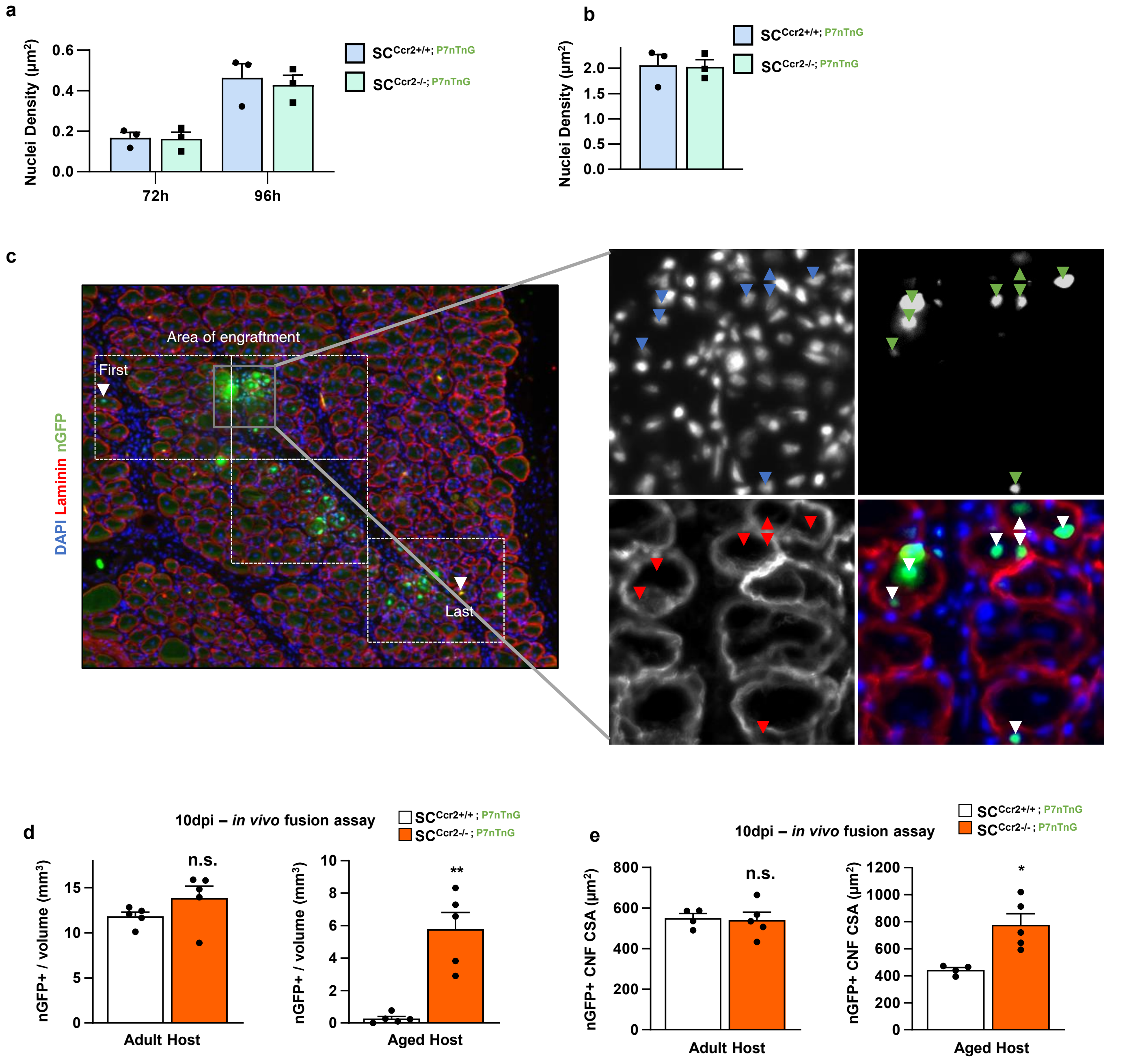

Supplementary Figure 8: Experimental strategy for *in vivo* fusion assay after satellite cell culture and transplant into regenerating muscle

a, Nuclei density of live adult SCs derived from Ccr2$^{+/+}$; Pax7$^{CreER/+}$; Rosa26$^{nTnG/+}$ (SC$^{Ccr2+/+ ; P7nTnG}$; n = 3) or Ccr2$^{-/-}$; Pax7$^{CreER/+}$; Rosa26$^{nTnG/+}$ (SC$^{Ccr2-/- ; P7nTnG}$; n = 3) culture after 72 hours by quantifying number of nGFP SCs per um^2. **b,** Nuclei density of remaining nGFP SCs post-trypsinization to assess survival derived from (a) (SC$^{Ccr2+/+ ; P7nTnG}$ n = 3; SC$^{Ccr2-/- ; P7nTnG}$; n = 3) . **c,** Representative picture for transplant analysis depicting the area of engraftment. nGFP-labelled SCs contributed to regeneration as depicted by GFP+ centrally nucleus in regenerating fibers. We used this method for all transplant experiments (n = 3 experiments). **d,** Quantification of absolute fused GFP+ nuclei normalized to host muscle volume (mm3) to assess transplant efficiency (n = 5 mice per experimental condition). **e,** Quantification of nGFP+ centrally nucleated fibers (CNF) size (μm2) to assess contribution of fused GFP+ cells to regeneration (n = 5 mice per experimental condition). Data are mean ± s.e.m. P < 0.05, **P < 0.01, ***P < 0.001, unpaired two-tailed Welch's t-test.

Supplementary Figure 9

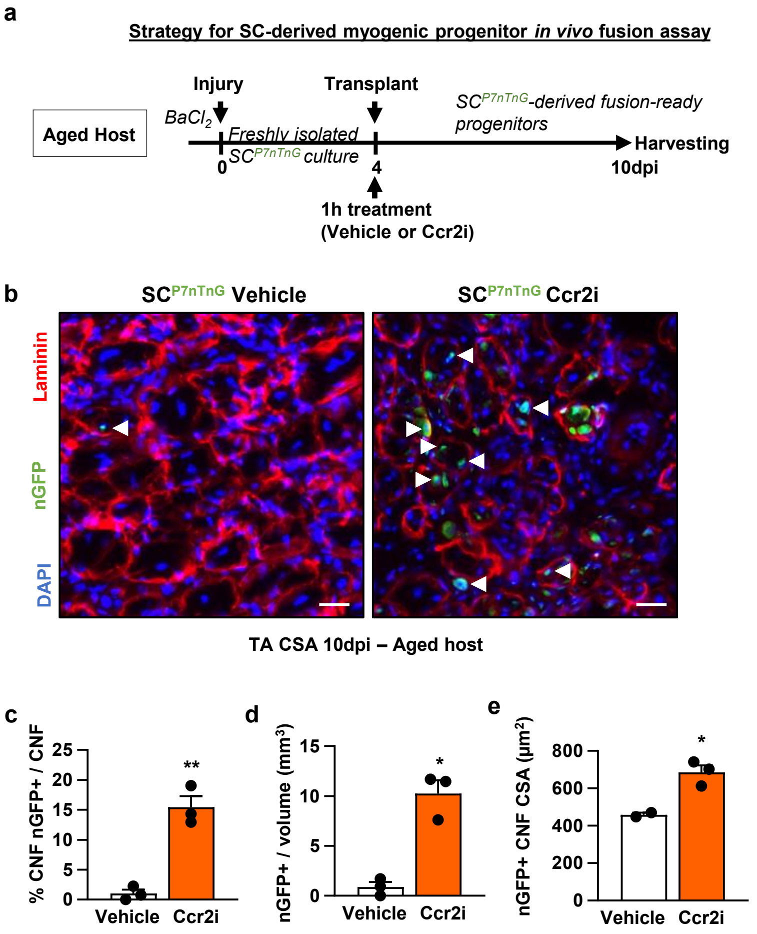

Supplementary Figure 9: Pharmacological inhibition of Ccr2 prior to transplant promotes satellite cell engraftment and fusion in aged regenerating host

a, Experimental strategy for freshly isolated SC culture to obtain *fusion-ready* myogenic progenitors prior to transplant and *in vivo* fusion assays. Adult SCs derived from Ccr2$^{+/+}$; Pax7$^{CreER/+}$; Rosa26$^{nTnG/+}$ (SC$^{Ccr2+/+;\ P7nTnG}$; $n = 3$) or Ccr2$^{-/-}$; Pax7$^{CreER/+}$; Rosa26$^{nTnG/+}$ (SC$^{Ccr2-/-;\ P7nTnG}$; $n = 3$) were isolated and cultured on recombinant extra cellular matrix gels to obtain fusion-competent progenitors prior to transplant. Cells were treated with Ccr2i for one hour as determined by the fusion assay (a,b). Cells were then washed, collected with Trypsin, washed again before being transplanted into aged regenerating hosts (4dpi). **b, c, (b)** Representative images and **(c)** quantification of regenerating aged ($n = 3$ mice per condition) muscles 6 days post-transplant (10dpi) for *in vivo* fusion assay. Experiment was performed once. **d,** Quantification of absolute fused GFP+ nuclei normalized to host muscle volume (mm^3) to assess transplant efficiency. **e,** Quantification of nGFP+ centrally nucleated fibers (CNF) size (µm^2) to assess contribution of fused GFP+ cells to regeneration. Scale bars, 25µm. Data are mean ± s.e.m. $P < 0.05$, $^{**}P < 0.01$, $^{***}P < 0.001$, unpaired two-tailed Welch's t-test.

Supplementary Figure 10

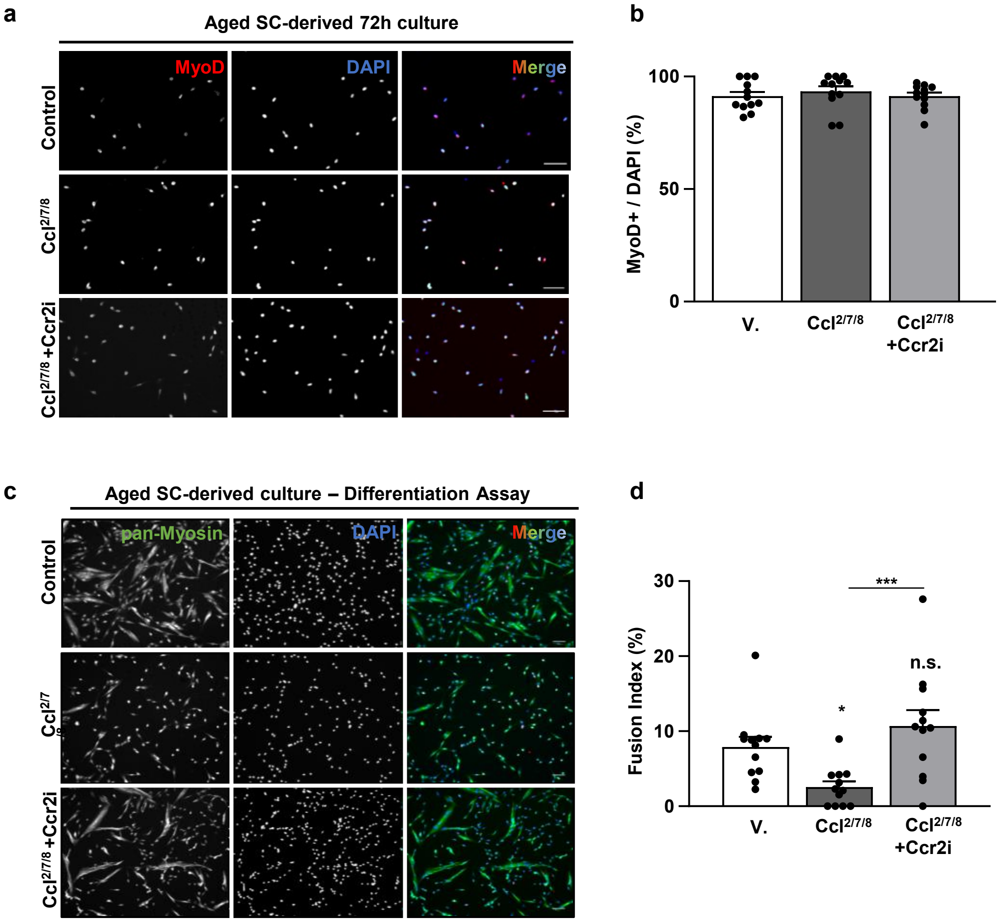

Supplementary Figure 10: Aged SCs are responsive to Ccr2 chemokines

a, Representative pictures of freshly isolated aged SCs cultured for 72 hours. **b,** Percentage of aged SC-derived myogenic progenitors ($MyoD^{+}$) after 72 hours of culture. n = 12 mice. Percentages are relative to total number of DAPI+ nuclei. **c, d, (c)** Immunostaining and **(d)** quantification of myogenic progenitor fusion capacity from aged SCs cultured for 96 hours followed by 24 hours treatment in low serum conditions. Cells were stained for myotube marker Myosin (green) and DAPI for nuclei (blue). n = 12 mice. Data are mean ± s.e.m. For each experiment >300cells, at least 2wells per condition were quantified. Scale bars, 50µm. n.s. not significant, *$P < 0.05$, **$P < 0.01$, ***$P < 0.001$, non-parametric one-way ANOVA followed by Tukey's multiple comparisons test.

Supplementary Figure 11

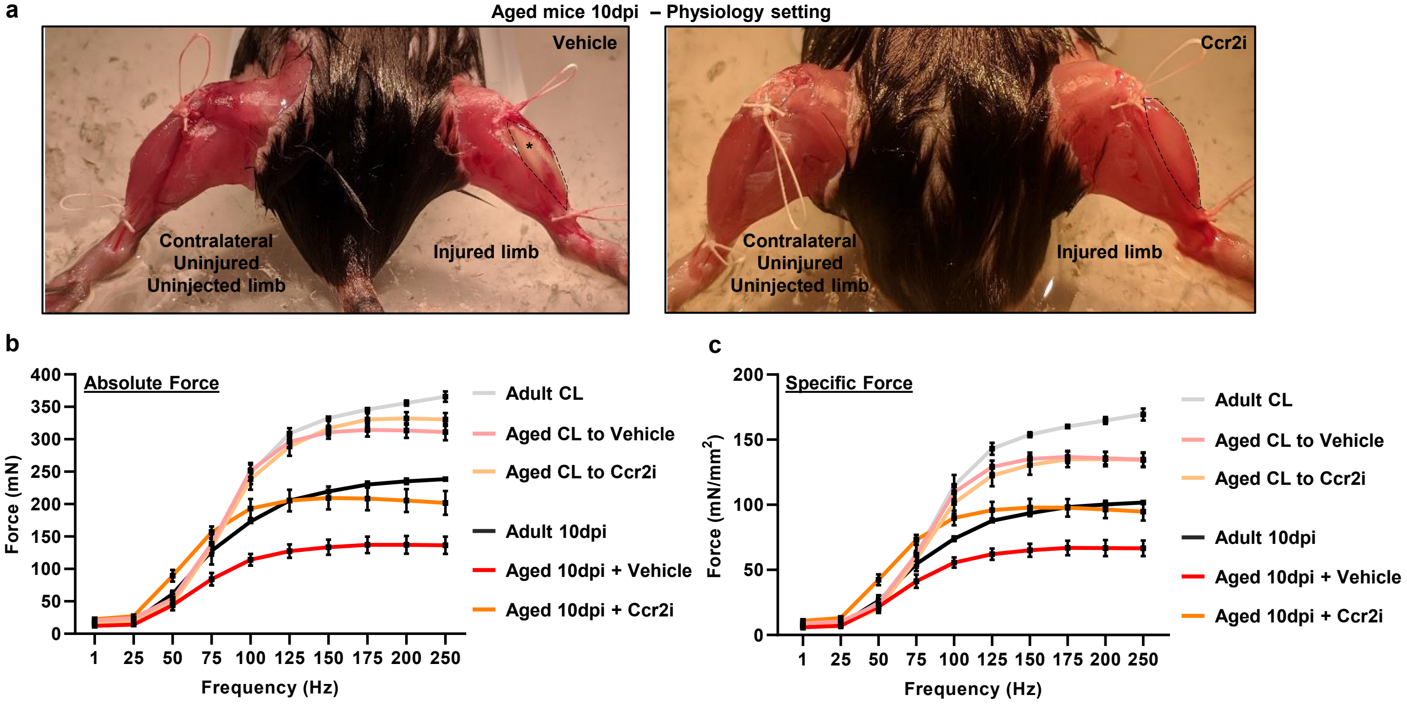

Supplementary Figure 11: Experimental design for muscle force generation

a, Representative pictures of aged injured tibialis anterior and extensor digitorum longus muscles (doted lines) at 10dpi, sutured prior to *ex vivo* physiology assay. The injured muscles treated with vehicle (left image) show discoloration characteristic of aged-associated fibrosis following impaired regeneration (*). This muscle was one of four muscles (out of height) that did not generate sufficient force for measurement. **b, c, (b)** Absolute and **(c)** specific force curves generated by EDL from adult (n = 3) and aged (n = 8 for each condition) mice. Data are mean ± s.e.m.

Supplementary Figure 12

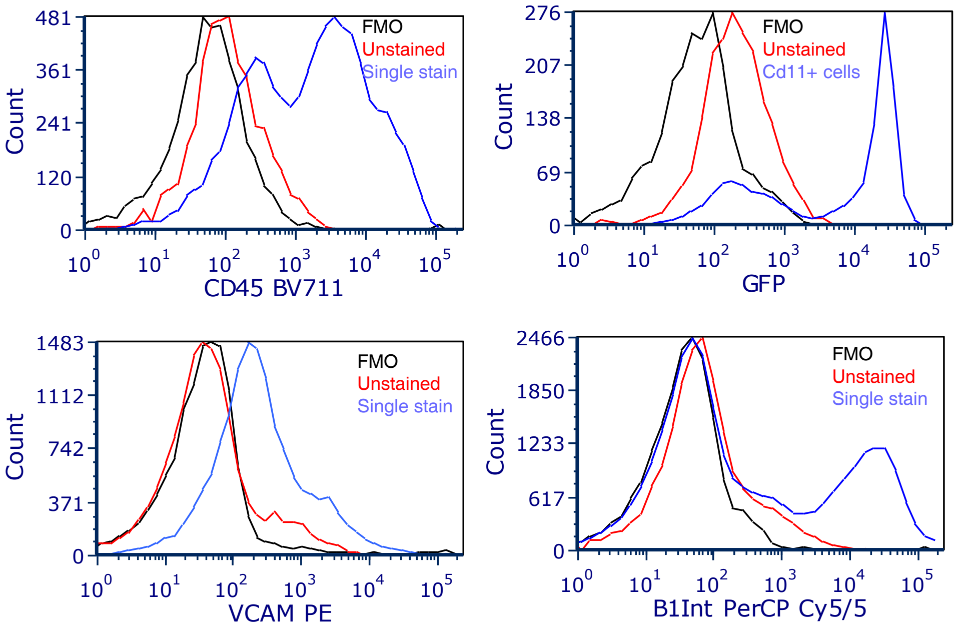

Supplementary Figure 12: Example of flow cytometry controls for antibodies used in cell sorting and analysis
