# Supplementary material for: Inhibition of inflammatory CCR2 signaling promotes aged muscle regeneration and strength recovery after injury
Source: Nat Commun. 2020 Aug 20;11:4167. doi: 10.1038/s41467-020-17620-8 (PMC7441393; doi:10.1038/s41467-020-17620-8)
Supplement: Supplementary file 2 — Reporting Summary [file 41467_2020_17620_MOESM2_ESM.pdf]

nature research

Corresponding author(s): Joe Chakkalakal

Last updated by author(s): Jun 8, 2020

Reporting Summary

Nature Research wishes to improve the reproducibility of the work that we publish. This form provides structure for consistency and transparency in reporting. For further information on Nature Research policies, see our Editorial Policies and the Editorial Policy Checklist.

Statistics

For all statistical analyses, confirm that the following items are present in the figure legend, table legend, main text, or Methods section.

n/a	Confirmed	
☐	☒	The exact sample size (*n*) for each experimental group/condition, given as a discrete number and unit of measurement
☐	☒	A statement on whether measurements were taken from distinct samples or whether the same sample was measured repeatedly
☐	☒	The statistical test(s) used AND whether they are one- or two-sided<br>*Only common tests should be described solely by name; describe more complex techniques in the Methods section.*
☐	☒	A description of all covariates tested
☐	☒	A description of any assumptions or corrections, such as tests of normality and adjustment for multiple comparisons
☐	☒	A full description of the statistical parameters including central tendency (e.g. means) or other basic estimates (e.g. regression coefficient) AND variation (e.g. standard deviation) or associated estimates of uncertainty (e.g. confidence intervals)
☐	☒	For null hypothesis testing, the test statistic (e.g. *F*, *t*, *r*) with confidence intervals, effect sizes, degrees of freedom and *P* value noted<br>*Give P values as exact values whenever suitable.*
☒	☐	For Bayesian analysis, information on the choice of priors and Markov chain Monte Carlo settings
☒	☐	For hierarchical and complex designs, identification of the appropriate level for tests and full reporting of outcomes
☒	☐	Estimates of effect sizes (e.g. Cohen's *d*, Pearson's *r*), indicating how they were calculated

Our web collection on statistics for biologists contains articles on many of the points above.

Software and code

Policy information about availability of computer code

Data collection	BD FACSDiva software (v8.0), Echo PRO software (v4.2), LI-COR Image Studio software (v3.1), StepOnePlus software (v2.3), BioRad BioPlex Manager software (v6.2),Image Lab (v6.0)
Data analysis	FCS Express 6 (v6.06.0021), GraphPad Prism 8 (v8.2.1), Fiji ImageJ (v2.0.0), Image Studio Light (V5.2)

For manuscripts utilizing custom algorithms or software that are central to the research but not yet described in published literature, software must be made available to editors and reviewers. We strongly encourage code deposition in a community repository (e.g. GitHub). See the Nature Research guidelines for submitting code & software for further information.

Data

Policy information about availability of data

All manuscripts must include a data availability statement. This statement should provide the following information, where applicable:

- Accession codes, unique identifiers, or web links for publicly available datasets
- A list of figures that have associated raw data
- A description of any restrictions on data availability

All data generated or analyzed during this study are included in this published article (and its supplementary information files).

Field-specific reporting

Life sciences study design

All studies must disclose on these points even when the disclosure is negative.

Sample size	Sample size was based on previous experiments, published work and associated power analysis. For this manuscript we did not perform additional power analysis. In some cases, data from independent experiments were combined and this was clearly stated in the figure legend.<br>• For flow cytometry experiments:<br>Paris, N.D., Soroka, A., Klose, A., Liu, W. & Chakkalakal, J.V. Smad4 restricts differentiation to promote expansion of satellite cell derived progenitors during skeletal muscle regeneration. eLife 5(2016).<br>Cosgrove BD, Gilbert PM, Porpiglia E, et al. Rejuvenation of the muscle stem cell population restores strength to injured aged muscles. Nat Med. 2014;20(3):255-264. doi:10.1038/nm.3464<br>• For muscle regeneration and transplant experiments:<br>Chakkalakal, J.V., Jones, K.M., Basson, M.A. & Brack, A.S. The aged niche disrupts muscle stem cell quiescence. Nature 490, 355-360 (2012).<br>Chakkalakal, J.V., et al. Early forming label-retaining muscle stem cells require p27kip1 for maintenance of the primitive state. Development 141, 1649-1659 (2014).<br>Zismanov V, Chichkov V, Colangelo V, et al. Phosphorylation of eIF2α Is a Translational Control Mechanism Regulating Muscle Stem Cell Quiescence and Self-Renewal. Cell Stem Cell. 2016;18(1):79-90. doi:10.1016/j.stem.2015.09.020<br>Cosgrove BD, Gilbert PM, Porpiglia E, et al. Rejuvenation of the muscle stem cell population restores strength to injured aged muscles. Nat Med. 2014;20(3):255-264. doi:10.1038/nm.3464<br>Price FD, von Maltzahn J, Bentzinger CF, et al. Inhibition of JAK-STAT signaling stimulates adult satellite cell function [published correction appears in Nat Med. 2014 Oct;(10):1217] [published correction appears in Nat Med. 2015 Apr;21(4):414]. Nat Med. 2014;20(10):1174-1181. doi:10.1038/nm.3655<br>• For culture, immunoblot and qPCR experiments<br>Zismanov V, Chichkov V, Colangelo V, et al. Phosphorylation of eIF2α Is a Translational Control Mechanism Regulating Muscle Stem Cell Quiescence and Self-Renewal. Cell Stem Cell. 2016;18(1):79-90. doi:10.1016/j.stem.2015.09.020<br>Price FD, von Maltzahn J, Bentzinger CF, et al. Inhibition of JAK-STAT signaling stimulates adult satellite cell function [published correction appears in Nat Med. 2014 Oct;(10):1217] [published correction appears in Nat Med. 2015 Apr;21(4):414]. Nat Med. 2014;20(10):1174-1181. doi:10.1038/nm.3655<br>Cosgrove BD, Gilbert PM, Porpiglia E, et al. Rejuvenation of the muscle stem cell population restores strength to injured aged muscles. Nat Med. 2014;20(3):255-264. doi:10.1038/nm.3464
Data exclusions	All data were included in the current study.
Replication	Flow cytometry using Ccr2 GFP mice was performed in triplicate. Luminex was replicated twice. Culture experiments were performed at least 4times, except culture with the Ccr2 mutants that was replicated one time. qPCR and immunoblots were performed at least three times at different occurrences using different samples. siRNA treatment and associated culture were replicated three times. Muscle injuries were replicated at least three times. Transplant and physiology experiments were performed once using a large cohort of mice.
Randomization	Mice were litter mates and randomly assigned to groups. All samples were derived from mice that were randomly assigned for treatments. All treatments, gene modifications, and appropriate controls for primary cell culture experiments were done together, and wells randomly assigned for treatments.
Blinding	All cell culture immunostaining analysis were performed in a double-blind manner. Fiber sizing images were acquired blinded and analysis was performed by automated image quantification. Blinding was not done for the other experiments, however note that FACS and Luminex were performed by independent core technicians who were unaware of the conditions and hypothesis. Specifically, experiments where blinding was possible were analyzed in a blinded manner. Other experiments were conducted by a person (core operator) that was aware that samples were different, but not privy to the hypothesis or had any vested interest in the conclusion.

Reporting for specific materials, systems and methods

We require information from authors about some types of materials, experimental systems and methods used in many studies. Here, indicate whether each material, system or method listed is relevant to your study. If you are not sure if a list item applies to your research, read the appropriate section before selecting a response.

Materials & experimental systems

n/a	Involved in the study	
☐	☒	Antibodies
☒	☐	Eukaryotic cell lines
☒	☐	Palaeontology and archaeology
☐	☒	Animals and other organisms
☒	☐	Human research participants
☒	☐	Clinical data
☒	☐	Dual use research of concern

Methods

n/a	Involved in the study	
☒	☐	ChIP-seq
☐	☒	Flow cytometry
☒	☐	MRI-based neuroimaging

Antibodies

Antibodies used	The following antibodies were used: Mouse anti-Pax7 (1:100, Developmental Studies Hybridoma Bank (DSHB), Iowa City, IA), mouse anti-MyoD (BD Biosciences #554130), mouse anti-phospho-MyoD (1:1000, Sigma) rabbit anti-Myogenin (1:250, AbCam), rat or rabbit anti-Laminin (1:1000 or 1:1500, Sigma-Aldrich, L0663 or L9393), rabbit anti-skeletal muscle myosin (1:250, Sigma-Aldrich HPA1239), rabbit or mouse anti-p38 (1:1000, Cell Signaling), rabbit or mouse anti-phospho-p38 (1:1000, Cell Signaling), rabbit anti-phospho-p38delta/gamma (1:1000, ThermoFisher), rabbit anti-Erk1/2 (1:1000, Cell Signaling), mouse anti-phospho-Erk1/2 (1:1000, Cell Signaling), VCAM (Biolegend), Beta1Integrin (Biolegend), CD45 (BD Science), CD31 (BD Science), Sca1 (Biolegend).<br>List of antibodies were also provided in the Experimental Procedure section of the manuscript.
Validation	For all antibodies we used the resource BenchSci (https://www.benchsci.com/)and CiteAb (https://www.citeab.com/) for validation, as well as current literature and manufacturer validation for the use, the species and cross-reactivity. Pax7 antibody (https://dshb.biology.uiowa.edu/PAX7) was validated by multiple sources and is the most widely used and cited Pax7 antibody in the literature. MyoD antibody (https://www.bdbiosciences.com/us/applications/research/stem-cell-research/mesoderm-markers/human/purified-mouse-anti-myod) only stained muscle progenitors and did not stained fused nuclei in myotubes. It was also validated by the manufacturer and used in 47 citations. Phospho-MyoD (https://www.sigmaaldrich.com/catalog/product/sigma/sab4503943?lang=en&region=US) and phospho-p38delta/gamma antibody (https://www.thermofisher.com/antibody/product/Phospho-p38-MAPK-gamma-delta-Tyr185-Tyr182-Antibody-Polyclonal/PA5-105907) was validated by manufacturer.Both were used and validated for ChIP and western blot in Gillespie et al., 2009 (doi: 10.1083/jcb.200907037). Myogenin antibody (https://www.abcam.com/myogenin-antibody-epr4789-ab124800.html) was validated in differentiating muscle stem cell cultures after differentiation used in 19 citations. Laminin antibodies (https://www.sigmaaldrich.com/catalog/product/sigma/l0663?lang=en&region=US) only stained the muscle fiber lamina and did not stain other cells or the muscle fibers. The rat antibody was used in 15 citations, while the rabbit was used in 985 citations. P38, phospho-p38, Erk and phospho-Erk antibodies were all validated by manufacturers Cell Signaling. We found time sensitive increase of phosphorylation in response to stimulation by known ligand FGF. All are widely cited in the literature (>1000 citations). All antibodies used in flow cytometry are from BD Sciences or BioLegend and routinely validated by manufacturers and are widely used in the literature.

Animals and other organisms

Policy information about studies involving animals; ARRIVE guidelines recommended for reporting animal research

Laboratory animals	All mice used in this study were male C57BL6 adult (4–6 months; Jackson Labs or NIA) and male aged (24–25months; NIA). For Ccr2-KO (Ccr2-/-) (004999) and Ccr2-KI/KO (Ccr2GFP/GFP) (027619) mice we used male mice ranging from 4-6months of age. Additionally, Pax7CreER/+; Rosa26nTnG/+ male mice 4 months were used (parental strains from Jackson Labs). Pax7CreER; Rosa26nTnG were breed with Ccr2-/- mice to generate Pax7CreER/+; Rosa26nTnG/+;Ccr2-/- and used for transplants at the age of 4months.
Wild animals	No wild animal were used in this study.
Field-collected samples	This study did not involved field-collected samples.
Ethics oversight	Animal experiments in this study were carried out in accordance with guidelines set by the Animal Care and Use Committee and protocols were reviewed and validated by the University of Rochester Committee on Animal Resources.

Note that full information on the approval of the study protocol must also be provided in the manuscript.

Flow Cytometry

Plots

Confirm that:

- [x] The axis labels state the marker and fluorochrome used (e.g. CD4-FITC).
- [x] The axis scales are clearly visible. Include numbers along axes only for bottom left plot of group (a 'group' is an analysis of identical markers).
- [x] All plots are contour plots with outliers or pseudocolor plots.
- [x] A numerical value for number of cells or percentage (with statistics) is provided.

Methodology

Sample preparation	For FACS, mice were euthanized with C02 following UCAR guideline. Skeletal muscles were harvested, chopped and dissociated using the Gentle MACS and dissociation buffer from manufacturer. Single cells suspensions were filtered with 70um SmartFilter filters and washed with F10 media. Lastly, cells were resuspended in PBS 0.5%BSA prior to staining and sort.
Instrument	All cell sorting was performed on BD FACSAria II machines model.
Software	All flow cytometry data and statistics were first acquired on native BD FACSDiva software, and then re-analyzed on FCS Express (v6.06.0021) for figure purposes.

Cell population abundance	We were able to assess purity by using multiple lineage negative and positive markers during the sort and confirmed purity in culture by immunostaining or qPCR analysis targeting cell-specific markers. Muscle stem cells (satellite cells) were sorted as Cd45- CD31- SCA1- CD11b- VCAM+ B1Int+ cells. 100% of sorted muscle stem cells were Pax7+ 24h after plating and MyoD+ 72h after plating.
Gating strategy	Gating was determined using FMO for each channels in each new FACS experiment to ensure that positive populations were specifically recognized by the antibody for that specific marker with no overlap and to re-adjust the machine voltage. FMO examples were provided in Extended Material.

☒ Tick this box to confirm that a figure exemplifying the gating strategy is provided in the Supplementary Information.
